# Supplementary material for: A Bidirectional Circuit Switch Reroutes Pheromone Signals in Male and Female Brains
Source: Cell. 2013 Dec 19;155(7):1610–23. doi: 10.1016/j.cell.2013.11.025 (PMC3898676; doi:10.1016/j.cell.2013.11.025)
Supplement: Table S1. Summary of Recorded Cells and All Statistical Tests in This Study, Related to Table 1 — (A) Total numbers of wild-type neurons recorded in odor stimulation experiments (n = 130), organized by cluster and driver. Only neurons that were successfully filled during recording are included in this study. (B) Total numbers of recorded neurons (n = 288), organized by cluster and genotype and sex. WT, wild-type; (1), DA1 stimulation experiments. (C) Summary of all statistical tests. [file mmc1.pdf]

Table S1. Summary of Recorded Cells and All Statistical Tests in This Study, Related to Table 1

A

cluster	driver	female	male
**aSP-f**	*fru*^{Gal4}	0	7
	JK1029	29	11
	JK56	8	20
**aSP-g**	*fru*^{Gal4}	0	0
	JK1029	16	17
	JK56	0	0
**aSP-h**	*fru*^{Gal4}	0	0
	JK1029	8	14
	JK56	0	0

B

cluster	genotype	female	male
**aSP-f**	*wt*	38	37
	Or67d$^{-/-}$	1	20
	DA1$^{(1)}$	1	11
	fru^{F}	0	14
	fru^{M}	18	0
	tra^{1}	14	0
**aSP-g**	*wt*	16	17
	Or67d$^{-/-}$	10	0
	DA1$^{(1)}$	9	8
	fru^{F}	0	0
	fru^{M}	17	0
	tra^{1}	9	0
**aSP-h**	*wt*	8	21
	Or67d$^{-/-}$	0	0
	DA1$^{(1)}$	2	0
	fru^{F}	0	0
	fru^{M}	9	0
	tra^{1}	8	0

C

**Sex-specific pheromone responses in *fru*+ LHNs**	**Cluster**	**Group 1**	**Group 2**	**Figure**	**Test**	**Hypothesis**	**Statistic**	**p**	**n**
Proportion cVA-responsive	aSP-f	female	male	1M	χ^2		χ^2=22.22	2.4e-06 ***	71
	aSP-g	female	male	1N	χ^2		χ^2=15.47	8.4e-05 ***	32
	aSP-h	female	male	1O	χ^2		χ^2=8.10	0.0044 **	22
Magnitude cVA response	aSP-f	female	male	1M	One way (permutation)	$\mu_1 \neq \mu_2$	Z=-4.02	5.7e-05 ***	71
	aSP-g	female	male	1N	One way (permutation)	$\mu_1 \neq \mu_2$	Z=3.79	0.00015 ***	32
	aSP-h	female	male	1O	One way (permutation)	$\mu_1 \neq \mu_2$	Z=-2.24	0.025 *	22
Compare Magnitude (cVA-responsive only)	aSP-f,aSP-g	aSP-g female	aSP-f male	1M–O	One way (permutation)	$\mu_1 \neq \mu_2$	Z=-2.20	0.028 *	33
Compare Sparseness (cVA-responsive only)	aSP-f,aSP-g	aSP-g female	aSP-f male	2G	Wilcoxon	$\mu_1 \neq \mu_2$	Z=3.62	3e-04 ***	45
Proportion cVA-responsive (unilateral vs bilateral)	aSP-f	aSP-f uni male	aSP-f bilat male	2H	χ^2		χ^2=7.94	0.0048 **	37
Compare Magnitude (unilateral vs bilateral)	aSP-f	aSP-f uni male	aSP-f bilat male	2H	One way (permutation)	$\mu_1 \neq \mu_2$	Z=3.93	8.3e-05 ***	37
**cVA responses in *fru*+ LHNs depend on a common input**									
Proportion cVA-responsive	aSP-f	wt male	*Or67d*$^{-/-}$ male	3C	χ^2		χ^2=8.44	0.0037 **	30
	aSP-g	wt female	*Or67d*$^{-/-}$ female	3C	χ^2		χ^2=13.10	3e-04 ***	25
Magnitude cVA response	aSP-f	wt male	*Or67d*$^{-/-}$ male	3C	One way (permutation)	$\mu_1 > \mu_2$	Z=3.12	0.00089 ***	30
	aSP-g	wt female	*Or67d*$^{-/-}$ female	3C	One way (permutation)	$\mu_1 > \mu_2$	Z=3.07	0.0011 **	25
**Fru^{M} is necessary for the male form of the switch**									
Magnitude cVA response	aSP-f	wt male	*fru*$^{-/-}$ male	4C	One way (permutation)	$\mu_1 > \mu_2$	Z=1.73	0.041 *	34
Compare Sparseness (cVA-responsive only)	aSP-f	wt male	*fru*$^{-/-}$ male	4D	Wilcoxon	$\mu_1 \neq \mu_2$	Z=1.84	0.066 .	11
**Fru^{M} specifies the male form of the circuit switch**									
Proportion cVA-responsive	aSP-f	wt female	*fru^{M}* female	5H	χ^2		χ^2=10.78	0.001 **	44
	aSP-g	wt female	*fru^{M}* female	5H	χ^2		χ^2=19.00	1.3e-05 ***	32
	aSP-h	wt female	*fru^{M}* female	S5C	χ^2		χ^2=0.70	0.4	17
Magnitude cVA response	aSP-f	wt female	*fru^{M}* female	5H	One way (permutation)	$\mu_1 < \mu_2$	Z=-2.97	0.0015 **	44
	aSP-g	wt female	*fru^{M}* female	5H	One way (permutation)	$\mu_1 > \mu_2$	Z=3.87	5.4e-05 ***	32
	aSP-h	wt female	*fru^{M}* female	S5C	One way (permutation)	$\mu_1 < \mu_2$	Z=-0.43	0.33	17
**Selectively masculinising *fru*+ LHNs can flip the switch**									
Proportion cVA-responsive	aSP-f	wt female	*tra^{1}* female	6H	χ^2		χ^2=9.74	0.0018 **	48
	aSP-g	wt female	*tra^{1}* female	6H	χ^2		χ^2=12.18	0.00048 ***	24
	aSP-h	wt female	*tra^{1}* female	S5G	χ^2		χ^2=4	0.046 *	16
Magnitude cVA response	aSP-f	wt female	*tra^{1}* female	6H	One way (permutation)	$\mu_1 < \mu_2$	Z=-2.51	0.0061 **	48
	aSP-g	wt female	*tra^{1}* female	6H	One way (permutation)	$\mu_1 > \mu_2$	Z=3.03	0.0012 **	24
	aSP-h	wt female	*tra^{1}* female	S5G	One way (permutation)	$\mu_1 < \mu_2$	Z=-2.06	0.02 *	16
