# Supplementary material for: A Bidirectional Circuit Switch Reroutes Pheromone Signals in Male and Female Brains
Source: Cell. 2013 Dec 19;155(7):1610–23. doi: 10.1016/j.cell.2013.11.025 (PMC3898676; doi:10.1016/j.cell.2013.11.025)
Supplement: Document S1. Article plus Supplemental Information [file mmc2.pdf]

Cell

A Bidirectional Circuit Switch Reroutes Pheromone Signals in Male and Female Brains

Johannes Kohl,[1,2] Aaron D. Ostrovsky,[1,2,3] Shahar Frechter,[1] and Gregory S.X.E. Jefferis[1,*]
[1]Division of Neurobiology, MRC Laboratory of Molecular Biology, Cambridge CB2 0QH, UK
[2]These authors contributed equally to this work
[3]Present address: Centre for Organismal Studies, Im Neuenheimer Feld 329, Heidelberg University, 69120 Heidelberg, Germany
*Correspondence: jefferis@mrc-lmb.cam.ac.uk
http://dx.doi.org/10.1016/j.cell.2013.11.025
This is an open-access article distributed under the terms of the Creative Commons Attribution License, which permits unrestricted use, distribution, and reproduction in any medium, provided the original author and source are credited.

SUMMARY

The *Drosophila* sex pheromone cVA elicits different behaviors in males and females. First- and second-order olfactory neurons show identical pheromone responses, suggesting that sex genes differentially wire circuits deeper in the brain. Using in vivo whole-cell electrophysiology, we now show that two clusters of third-order olfactory neurons have dimorphic pheromone responses. One cluster responds in females; the other responds in males. These clusters are present in both sexes and share a common input pathway, but sex-specific wiring reroutes pheromone information. Regulating dendritic position, the *fruitless* transcription factor both connects the male-responsive cluster and disconnects the female-responsive cluster from pheromone input. Selective masculinization of third-order neurons transforms their morphology and pheromone responses, demonstrating that circuits can be functionally rewired by the cell-autonomous action of a switch gene. This bidirectional switch, analogous to an electrical changeover switch, provides a simple circuit logic to activate different behaviors in males and females.

INTRODUCTION

Many species exhibit sexually dimorphic behaviors, typically as part of their reproductive repertoire. These behaviors, which often have a substantial unlearned component, provide highly tractable paradigms to explore the genetic and neural circuit basis of behavior (Baker et al., 2001; Dickson, 2008; Dulac and Kimchi, 2007; Wu and Shah, 2011). As potent releasers of specific dimorphic behavior, sex pheromones are particularly experimentally advantageous (Wyatt, 2003; Touhara and Vosshall, 2009). Nevertheless, even here the neural mechanisms underlying differential processing within the brain remain largely unknown (Stowers and Logan, 2010).

Several models have been proposed for how pheromones can elicit different behavior in males and females. One model is exemplified by classic work on the attraction of male silkmoths to bombykol (Touhara and Vosshall, 2009). Here, one sex expresses a pheromone receptor, while the other is blind to this cue. However, this peripheral change cannot account for situations in which a common stimulus produces behavior in both sexes. These are likely due to circuit differences within the brain. For instance, in mice, only males show courtship behavior toward females, but after ablation of the vomeronasal organ females show female-directed courtship (Kimchi et al., 2007). This leads to a second model in which both sexes can express male behaviors, but these are normally repressed in females by sex-specific circuits downstream of pheromone detection. However, these circuit differences remain unknown, because the relevant receptors and downstream pathways have yet to be identified.

A simpler paradigm is offered by analogous results in flies and mice, in which a monomolecular pheromone can activate identified sensory neurons in both sexes (Kurtovic et al., 2007; Haga et al., 2010). In the mouse, the male pheromone ESP1 activates V2Rp5 sensory neurons in both sexes but produces distinct patterns of immediate-early gene expression in deeper brain nuclei (Haga et al., 2010). ESP1 triggers lordosis in females, but no effect on male behavior has been reported.

In *Drosophila*, the male pheromone 11-*cis*-vaccenyl acetate (cVA) stimulates courtship in females but decreases courtship and increases aggression in males (Kurtovic et al., 2007; Wang and Anderson, 2010). Because both first- and second-order olfactory neurons show similar cVA responses in males and females (Kurtovic et al., 2007; Datta et al., 2008), it is likely that some circuit difference deeper in the brain results in sex-specific behavioral output. Two further studies have characterized downstream elements of this pathway. Ruta et al. (2010) used an elegant tracing approach based on sequential photoactivation of green fluorescent protein to identify candidate third- and fourth-order neurons, some of which were shown to be cVA responsive in males. However, they were unable to characterize

these neurons anatomically or functionally in females, so the presence or nature of any circuit dimorphism remained unclear. In a parallel study, Cachero et al. (2010) used a genetic mosaic technique to carry out an exhaustive analysis of sexually dimorphic neurons in male and female brains. In the olfactory system they found two groups of third-order neurons, present in both sexes, that appeared to be differentially connected, suggesting a precise circuit hypothesis for differential pheromone processing in male and female brains (Figure 1A).

We now combine targeted in vivo whole-cell electrophysiology, high-resolution neuroanatomy, and genetic analysis to analyze cVA processing in male, female, and sex mosaic flies. We first establish a simple but efficient circuit motif: a bidirectional (or changeover) switch in which a common input is routed to different active outputs in each sex. We then demonstrate that the *fruitless* gene sets the state of this switch, specifying both the dendritic placement and pheromone responses of third-order olfactory neurons in a cell-autonomous manner.

RESULTS

Sex-Specific Pheromone Responses in *fru*+ LHNs

cVA processing in the first three layers of the fly olfactory system provides an ideal model to investigate the logic of neural circuit switches. Or67d olfactory receptor neurons (ORNs) are narrowly tuned to cVA and send axons to the DA1 glomerulus in the brain, where they synapse with DA1 projection neurons (PNs) (Couto et al., 2005; Fishilevich and Vosshall, 2005; Ha and Smith, 2006; Kurtovic et al., 2007; Schlief and Wilson, 2007). First-order ORNs and second-order PNs both express the terminal sex determination gene *fruitless* (henceforth ***fru+*** neurons) but appear functionally isomorphic (Kurtovic et al., 2007; Datta et al., 2008). Recent anatomical work on *fru*+ neurons (Cachero et al., 2010; Yu et al., 2010; Ruta et al., 2010) has identified five clusters of candidate third-order neurons of the lateral horn that may receive cVA pheromone information (summarized in Table 1). Each cluster descends from a different neuroblast (neural stem cell) (Cachero et al., 2010). Cachero et al. (2010) highlighted two neuronal clusters that were present in both sexes but had dendrites in sex-specific locations: aSP-f neurons had dendritic overlap with DA1 PN axon terminals in males, but not females, whereas aSP-g dendrites overlapped in females, but not in males (Figure 1B). Although suggestive, these purely anatomical results provided no functional evidence for a wiring difference that altered pheromone processing.

Ruta et al. (2010) also characterized aSP-f (DC1) neurons in males. However, negative anatomical observations led to a conclusion that these neurons were absent in females. Critically, Ruta et al. (2010) then demonstrated that male aSP-f/DC1 neurons receive input from the DA1 glomerulus and respond to cVA in males. However, in the absence of positive anatomical data or physiological recordings in females, it remained unclear whether these responses were sex-specific. Furthermore, this study did not identify neurons that might selectively receive pheromone information in females.

Our anatomical data (Cachero et al., 2010) prompted us to make in vivo recordings from *fru*+ lateral horn neurons (LHNs) in males and females. We obtained stable whole-cell patch clamp recordings (most >1 hr), giving access to subthreshold responses and morphology of every recorded neuron. Cells were filled, classified, reconstructed (Evers et al., 2005) (see Figure 1C), and registered to a template brain (Cachero et al., 2010), allowing us to compare the overlap of LHN dendrites with incoming PN axons. One important technical point quickly became clear: fru^{Gal4} is too weakly expressed in females to target some cells for recording (e.g., aSP-f neurons). However, in the course of a large enhancer trap screen (S.F., J.K., and G.S.X.E.J., unpublished data; Experimental Procedures), we obtained two new driver lines, *JK1029* and *JK56*, that label subsets of *fru*+ neurons, including the aSP-f cluster, in both sexes (Figure S1 available online).

Because aSP-f dendrites only overlap with DA1 PNs in males (Figure 1D), we expected them to show male-specific responses. Indeed, about half of the male—but almost no female—aSP-f neurons showed significant spiking cVA responses (Figure 1M). cVA-responsive aSP-f neurons in males were narrowly tuned to cVA (Figures 1J and 2G), matching the narrow tuning of Or67d ORNs and DA1 PNs (Ha and Smith, 2006; Schlief and Wilson, 2007).

Because aSP-g dendrites overlap with DA1 PNs only in females (Figure 1E), we expected them to show female-specific responses. Indeed, the majority of female—but almost no male—aSP-g neurons responded to cVA (Figure 1N). Female aSP-g neurons showed weaker cVA responses and broader odor tuning than did male aSP-f neurons (Table S1C; Figures 1K and 2G); this is likely due to the partial overlap of aSP-g dendrites with incoming DA1 PN axons and suggests that their dendrites also receive information from other PN classes.

We also recorded from a third cluster of *fru*+ LHNs. aSP-h neurons were examined anatomically in males by Ruta et al. (2010) (who referred to them as DC2 neurons and proposed that they were absent from females), whereas Cachero et al. (2010) examined both sexes and observed a difference in the density of dendritic arbors in the ventral lateral horn (see Table 1). Examining single aSP-h neurons filled during recording, we found more dendritic overlap with DA1 PN axons in males than in females (Figures 1F and 1I). Functionally, cVA spiking responses in these broadly tuned neurons were stronger and more frequent in males but were still occasionally present in females (Figures 1L, 1O, and 2C). Given the quantitative nature of this difference and the broader tuning of these neurons, our subsequent analysis focused on aSP-f and aSP-g neurons.

Morphological and Functional Correlations

The difference in dendritic location for male and female aSP-f neurons (Figure 1D) provides a simple circuit hypothesis for the origin of functional differences between these neurons. We confirmed this relationship by examining the three-dimensional (3D) morphology of 37 male aSP-f neurons and 36 female aSP-f neurons that were filled during recording. Almost all male neurons had dendrites in the ventral lateral horn, whereas female neurons never did. In addition to clear differences in dendritic arborization, there was a consistent difference in axonal morphology. Male aSP-f axons terminate in the male-enlarged arch and lateral junction neuropil regions (Cachero et al., 2010; Yu et al., 2010), whereas female aSP-f axons project to the arch and the superior protocerebrum (Figure 2D). aSP-g and

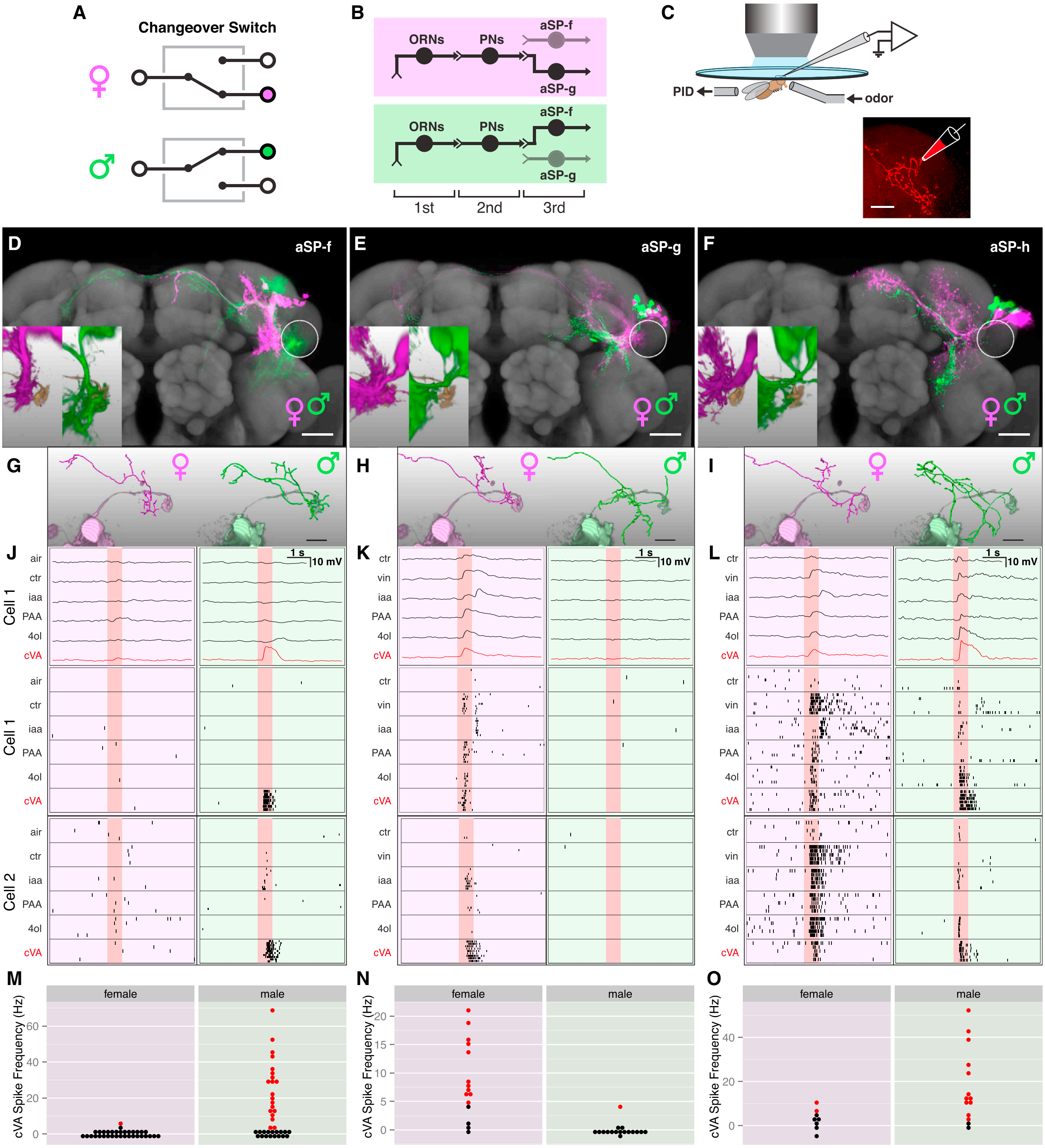

Figure 1. Sex-Specific Pheromone Responses in *fru*+ LHNs

(A and B) Abstract circuit model for sexually dimorphic behavior (A), and circuit model for cVA processing in females and males (B).

(C) Targeted in vivo whole-cell recording setup, with odor delivery and photoionization detector (PID). A dye-filled neuron is shown.

(D–F) Z projections of female and male neuroblast clones on a reference brain; the ventral lateral horn is marked with a white circle. Insets show spatial relationship between LHN dendrites and DA1 PN axon terminals (ochre). Cell numbers for cluster aSP-f: 23.2 ± 2.6 in males versus 18.6 ± 5.0 in females; aSP-g: 13.4 ± 0.89 versus 13.4 ± 4.97; aSP-h: 5.0 ± 0.8 versus 5.0 ± 0.5.

(G–I) Single aSP-f, aSP-g, and aSP-h LHNs filled during patch-clamp recording and traced (magenta or green lines) compared with volume-rendered DA1 PNs (pale magenta or pale green).

(legend continued on next page)

Table 1. Summary of Studies of *fru+* LHNs

Cachero et al. (2010)		aSP-f	aSP-g	aSP-h	aSP-k	aIP-e
Cell Number	male	23.2 (2.6)	13.4 (0.9)	5.0 (0.8)	29.2 (3.3)	27.0 (4.2)
	female	18.6 (5.0)	13.4 (4.9)	5.0 (0.5)	20.2 (3.5)	27.0 (2.2)
Overlap DA1	male	+++	–	+	note 1	++
	female	–	++	±	note 1	++
PA-GFP Prediction	male	yes	no	yes	yes	yes
	female	no	yes	note 2	yes	yes
**Ruta et al. (2010)**		**DC1**	**n/a**	**DC2**	**LC1**	**LC2**
Cell Number	male	19.7 (2.3)	n/a	Note 3	25.8 (3.4)	13.0 (2.8)
	female	n/a	n/a	n/a	15.8 (3.0)	13.3 (2.1)
PA-GFP Observed	male	yes	no	yes	yes	yes
	female	no	no	no	yes	yes
DA1 Stim. Response	male	+++	n/a	–	+++	±
	female	n/a	n/a	n/a	n/a	n/a
cVA Response	male	+++	n/a	n/a	n/a	n/a
	female	n/a	n/a	n/a	n/a	n/a
**This Study**		**aSP-f**	**aSP-g**	**aSP-h**		
cVA Response	male	+++	–	+++		
	female	–	++	+		
DA1 Stim. Response	male	+++	–	n/a		
	female	n/a	++	n/a		
Cell Number fru^M Female		24.5 (0.8)	13.0 (0)	5.0 (0.5)		
Cell Number tra^1 Female		23.7 (1.4)	13.3 (0.7)	5.0 (0.8)		
Cell Number *JK1029*	male	18.2 (1.7)	11.2 (1.0)	5.0 (0.4)		
	female	12.8 (1.6)	11.3 (0.9)	5.0 (0.4)		
	fru^M female	18.8 (1.5)	11.7 (1.2)	5.0 (–)		
Cell Number *JK56*	male	6.4 (1.0)	5.6 (0.8)			
	female	6.8 (0.9)	5.7 (1.5)			
	fru^M female	5.6 (1.0)	5.5 (0.9)			
	$fru^{-/-}$ male	7.0 (1.1)	5.7 (0.6)			

Summary of *fru+* LHN clusters characterized in Cachero et al. (2010), Ruta et al. (2010), and this study. Cell numbers are given as mean (SD). cVA responses (DA1 overlap) range from very strong (+++) to absent (–). Discrepant results are underlined. We used the nomenclature of Cachero et al. (2010), which defines clusters of developmentally related groups of *fru+* lateral horn neurons, because this is established for all three clusters of lateral horn neurons studied (in both sexes); furthermore, the neuroblast of origin is a biologically invariant property rather than an experimental procedure (photoactivation, see Ruta et al. 2010), which may be somewhat variable. Note 1: aSP-k clones generated at larval hatching are missing some neurons with extensive dendritic arbors in the lateral horn. Compare with cluster aSP8 in Yu et al. (2010). Note 2: We predict that the level of PA-GFP labeling depends on a number of factors, including the strength of driver expression in the candidate postsynaptic neurons and the extent of their dendritic arbors in the vicinity of DA1 axons. The relatively weak overlap of DA1 PNs and aSP-h dendrites might not generate any PA-GFP signal. Note 3: Ruta et al. (2010) identified DC2/aSP-h in males but did not report cell counts. Cell counts (mean [SD]) for dimorphic LHN clusters labeled by *JK1029* and *JK56* hemidrivers when crossed to *Cha-Gal4-DBD*. Note *JK56* does not label aSP-h neurons. See also Table S1.

aSP-h neurons show a similar axonal dimorphism with axons in females targeting the same superior protocerebral region (Figures 2E and 2F). This region is the female-enlarged neuropil described by Cachero et al. (2010) that appears to match a focus for female receptivity described by early gynandromorph studies (Tompkins and Hall, 1983).

(J–L) Physiological data for aSP-f, aSP-g, and aSP-h LHNs. These three panels are arranged in a 3-row × 2-column grid. The top row shows averaged current clamp recordings for each LHN shown in (G), (H), and (I) (cell 1). Row 2 shows raster plots for the same neurons. Row 3 shows raster plots for an additional neuron (cell 2).

(M–O) Summary of cVA responses. Each dot is one neuron, colored red for significant cVA responses (adjusted $p < 0.01$, see the Experimental Procedures); nonsignificant responses are black. Response counts: aSP-f: 1/34 female and 20/37 male neurons; aSP-g: 11/15 female and 1/17 male neurons; aSP-h: 2/8 female and 12/14 male.

See Table S1C for statistics. Scale bars, 25 μm. Pale red bars in (J)–(L) mark 500 ms odor presentation. See the Experimental Procedures for odorant abbreviations and Figure S1 for additional data.

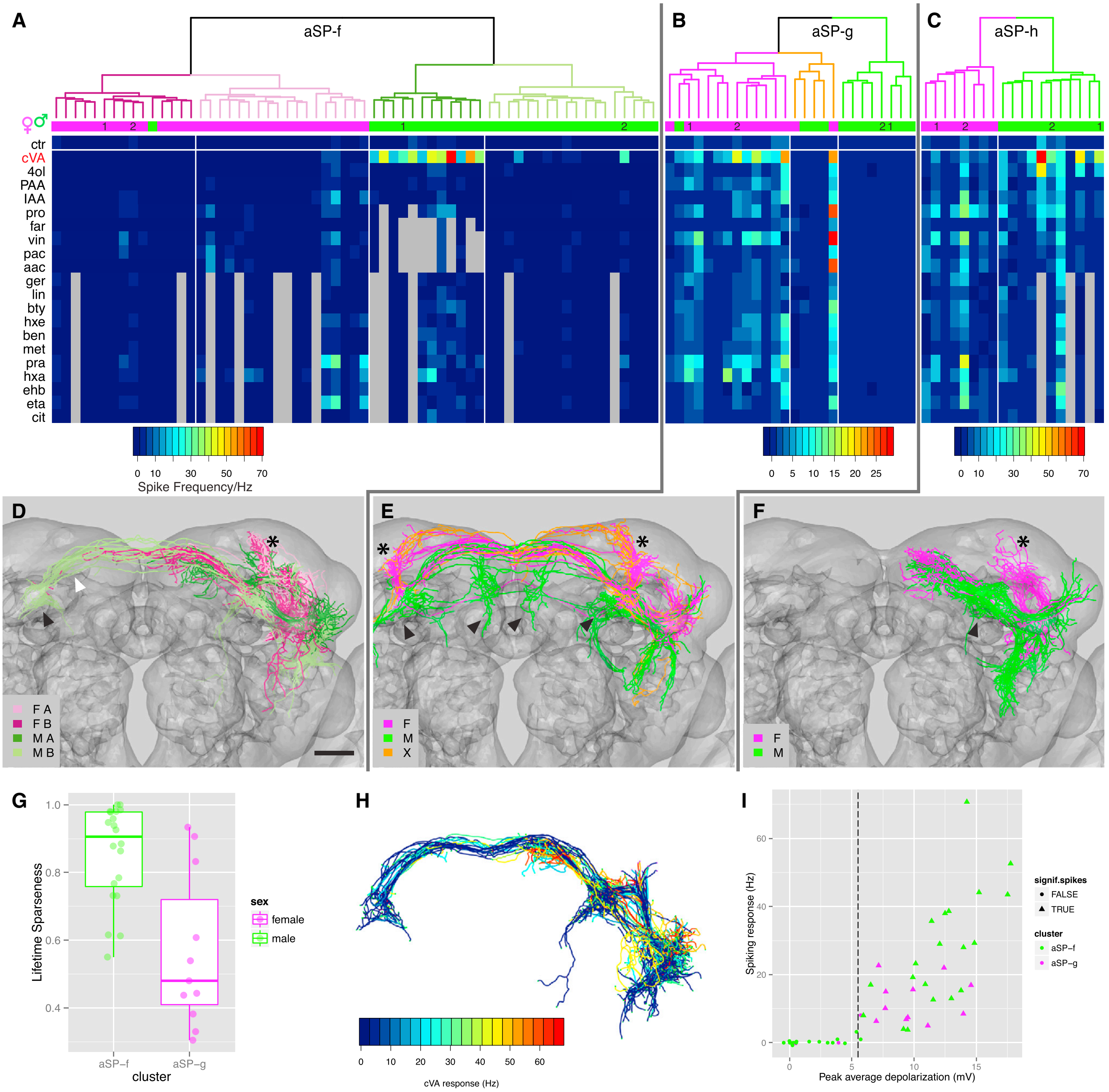

Figure 2. Morphological and Functional Correlations in *fru*+ LHNs

(A–C) Mean odor responses of aSP-f, aSP-g, and aSP-h LHNs displayed as heatmap. Data are ordered by a dendrogram of morphological similarity between each neuron at the top of the panel. Dendrograms are split into colored subclusters. Below each dendrogram, one row indicates the sex of each neuron. Note the very strong correspondence between morphological clusters and sex for all LHN classes. Physiological data are presented in a heatmap: each column is a single neuron, and each row represents an odorant. Each box represents the color-coded average spike frequency of a median of six odor trials. Gray boxes indicate untested odorants. Neurons displayed in Figures 1J–1L (cells 1–2) are numbered (1–2) in the first row.

(D–F) 3D renderings of morphological clusters identified in (A)–(C). Each panel shows all neurons from the heatmap above. Cells are color-coded according to dendrogram clusters in (A)–(C). The sex of neurons in each morphological cluster is extremely homogeneous, but note in (D) that strong cVA responders in aSP-f male cluster MA are entirely unilateral with stereotyped morphology and dendrites in the ventral lateral horn. Note in (E) that cluster X is not well resolved into distinct male and female groups. Asterisks and arrowheads in (D)–(F) mark female- and male-specific projections in the superior protocerebrum, respectively. In (D), a black arrowhead marks the lateral junction, and a white arrowhead marks the arch (see text).

(G) Lifetime sparseness (S) of male aSP-f neurons and female aSP-g neurons (see the Experimental Procedures). Male aSP-f neurons have significantly narrower odor tuning than do female aSP-g neurons (see Table S1C for details). Box plot rectangles cover the interquartile range (IQR); the median is marked by a hinge. Whiskers include all points within 1.5 × IQR of the hinge.

(legend continued on next page)

The lack of spiking responses in half of the male aSP-f neurons (Figure 1M) was initially surprising, because all but one of these nonresponders had dendrites in the ventral lateral horn (see Figure S2A) with the potential to form synapses with DA1 PNs. However, 9/17 of these neurons showed significant subthreshold cVA responses (see Extended Experimental Procedures), indicating that they do receive input, but that it is unable to drive a spiking response. Morphological analysis of aSP-f neurons revealed two major classes in males, unilateral neurons and bilateral neurons, whose axons project through the arch to the contralateral protocerebrum (Figures 2A and 2D). Intriguingly, cross-comparison of morphology and physiology revealed that all unilateral neurons in our study showed strong spiking responses, whereas responses from bilateral neurons were infrequent and weaker when present (Figures 2A and 2H; see Table S1C). aSP-f neurons therefore have distinct functionally and morphologically related subtypes. Furthermore, these subtypes are genetically heterogeneous, because the *JK56* driver line exclusively labels bilateral neurons. This difference may be functionally significant because bilateral male aSP-f neurons have additional dendritic arborizations ventral to the lateral horn (Figure 2H) and may therefore integrate both olfactory and nonolfactory stimuli; coincident inputs would likely result in stronger responses.

Analysis of individual aSP-g (Figures 2B and 2E) and aSP-h (Figures 2C and 2F) neurons clearly revealed the correlated morphological and functional differences between the sexes. However, although aSP-g neurons showed clear morphological subtypes within each sex (Figures 2B and 2E), no strong structure/function correlations were obvious for these subtypes. aSP-h neurons appeared morphologically homogeneous (Figures 2C and 2F).

cVA Responses in *fru*+ LHNs Depend on a Common Input

Our bidirectional switch model (Figure 1A) predicts that pheromone responses depend on a common sensory pathway. cVA detection has been linked to two classes of sensory neurons that express either olfactory receptor: Or67d or Or65a (Ha and Smith, 2006; van der Goes van Naters and Carlson, 2007; Kurtovic et al., 2007; Ejima et al., 2007). However, the available anatomical data suggest that aSP-f neurons in males (Cachero et al., 2010; Ruta et al., 2010) and aSP-g neurons in females (Cachero et al., 2010) are postsynaptic to DA1 PNs, which receive input from Or67d sensory neurons (Kurtovic et al., 2007; Schlief and Wilson, 2007). We therefore recorded from *fru*+ LHNs in flies lacking Or67d (Kurtovic et al., 2007) (Figure 3D). In these *Or67d*$^{-/-}$ flies, cVA-evoked spiking and subthreshold responses were abolished in both male aSP-f (Figures 3A and 3C) and female aSP-g neurons (Figures 3B and 3C; Table S1C). The absence of even subthreshold cVA responses indicates that Or65a ORNs provide minimal, if any, input to these neurons. Responses of female aSP-g neurons to other odorants were preserved in *Or67d*$^{-/-}$ flies (Figure 3B). This suggests that female aSP-g neurons integrate cVA information from the Or67d/DA1-labeled line along with general odor information encoded by other ORN/PN classes. In conclusion, the same Or67d sensory pathway is necessary for cVA responses in both aSP-f and aSP-g LHNs, consistent with the bidirectional switch hypothesis.

DA1 PNs Form Sex-Specific Connections with *fru*+ LHNs

The Or67d receptor is necessary for pheromone responses in *fru*+ LHNs. Is stimulating this pathway also sufficient to excite these neurons? Or67d sensory neurons project to the DA1 glomerulus, synapsing with DA1 PN dendrites. We used local acetylcholine iontophoresis (Ruta et al., 2010) to stimulate the dendrites of DA1 PNs, while simultaneously recording intracellularly from *fru*+ LHNs (Figure 3E). DA1 stimulation produced both spiking responses and large depolarizations in all male aSP-f and almost all female aSP-g neurons (Figures 3F–3H; Figure S3A); male aSP-g neurons were unresponsive. Control stimulation in neighboring glomeruli produced minimal responses (Figures 3F and 3H), confirming the specificity of stimulation; this also suggests that glomeruli in more distant parts of the antennal lobe are the origin of non-cVA responses in female aSP-g neurons. We previously noted that only half of aSP-f neurons showed cVA spiking responses, whereas all male aSP-f neurons responded to glomerular stimulation. This suggests that all aSP-f neurons receive input from DA1 PNs, but the strength of this input varies across different morphological classes. It also appears that stimulation can reveal functional connections that are too weak to generate spiking responses to odor stimuli.

The Or67d/DA1 pathway is sufficient for sex-specific excitation of aSP-f and aSP-g LHNs, but do these LHNs receive direct input from DA1 PNs? We measured the latency between presynaptic stimulation and postsynaptic response. Latencies to the first spike were variable though sometimes as low as 4.5 ms. However, whole-cell recordings allowed us to measure the latency to the start of the evoked postsynaptic response (see Figure S3B; Experimental Procedures) in male aSP-f and female aSP-g neurons. We found values of 1.8 ± 0.4 ms and 1.8 ± 0.3 ms (n = 7 each), respectively. This is less than half the reported latency for ORN to PN connections (Kazama and Wilson, 2008) but is consistent with measurements from paired recordings of connected nonpheromonal PNs and LHNs (1.5 ms; Fisek and Wilson, 2013).

Our recordings therefore provide conclusive evidence for sex-specific input from DA1 PNs to aSP-f and aSP-g LHNs, exactly as predicted from their anatomy and odor responses. Furthermore, the very short latency to subthreshold response is strong evidence for a monosynaptic connection.

(H) Color-coded spiking responses of male aSP-f neurons to cVA. Unilateral aSP-f neurons show strong responses (warm colors); bilateral neurons show weak responses (cold colors).

(I) Relationship between input (subthreshold response) and output (spikes) in response to cVA stimulation. Dashed line marks a threshold for the peak averaged depolarization of ~5.5 mV, above which the cell robustly fires action potentials. Cells showing a statistically significant spiking response are plotted as triangles. Note some aSP-f neurons show significant subthreshold responses without spiking.

Scale bar, 25 μm (D). See also Figure S2.

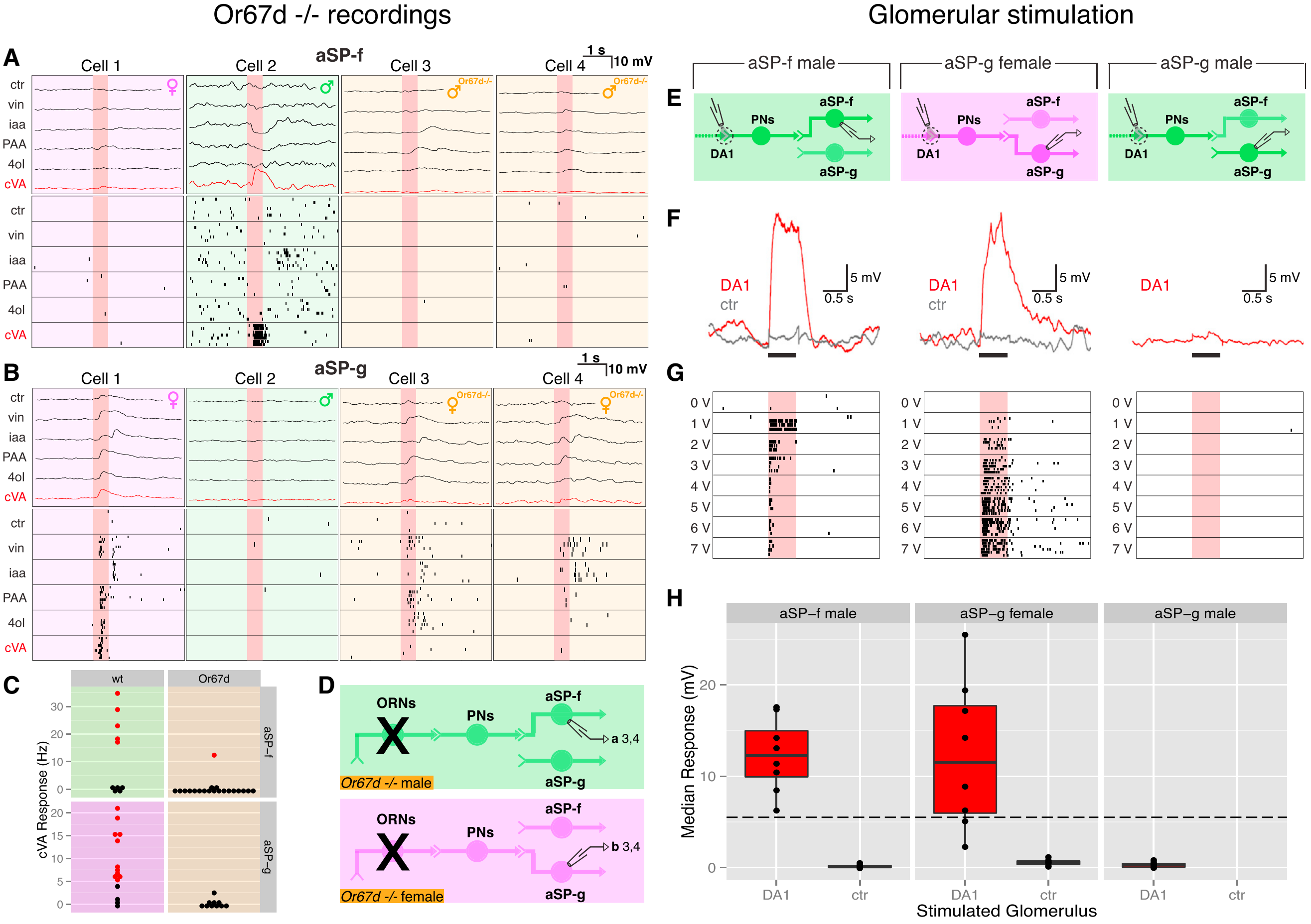

Figure 3. cVA Responses in *fru*+ LHNs Depend on a Common Input, and DA1 PNs Form Sex-Specific Connections with *fru*+ LHNs

(A and B) Physiological data for aSP-f and aSP-g LHNs. In each case, data for four neurons are shown: wild-type female, male, and two *Or67d* $^{-/-}$ animals. The top row shows averaged current clamp recordings; row 2 shows raster plots for the same neurons.

(C) cVA responses are abolished in *Or67d* $^{-/-}$ male aSP-f and *Or67d* $^{-/-}$ female aSP-g neurons. Each dot represents a neuron, colored red for significant cVA response (Experimental Procedures) or is black otherwise. See Table S1C for statistical analysis.

(D) Circuit models for *Or67d* $^{-/-}$ male and female brains. Labels refer to cells in (A) or (B).

(E) Circuit models for recording configuration of male aSP-f (left), female aSP-g (middle), and male aSP-g (right) neurons during glomerular stimulation. Dashed circle marks DA1 glomerulus.

(F and G) Physiological data for recordings of wild-type male aSP-f (left), female aSP-g (middle), and male aSP-g (right) LHNs during glomerular stimulation. (F) Single current clamp voltage responses to 3 V stimulation of DA1 or control (ctr) glomerulus. Black bar marks stimulation window. (G) LHN spiking responses scale with stimulation voltage.

(H) LHN depolarizations evoked by stimulation of DA1 or control glomerulus (male aSP-f neurons 12.3 ± 1.4 mV, mean ± SEM versus 0.1 ± 0.1 mV in control [n = 11]; female aSP-g neurons 12.3 ± 2.8 mV versus 0.6 ± 0.1 mV in control [n = 9, of which eight were responsive]; n = 8 male aSP-g recordings). Note control stimulation was not performed in unresponsive male aSP-g neurons. Box plot rectangles mark the interquartile range (IQR); the median is marked by a black hinge. Whiskers include points within 1.5 × IQR of the hinge. Dashed line marks threshold of ~5.5 mV, above which spikes are reliably observed (see Figure 2I). Pale red bars in (A), (B), and (G) mark 500 ms odor presentation. See also Figure S3.

Fru^{M} Is Necessary for the Male Form of the Switch

Our results so far identify a bidirectional switch in pheromone processing (Figure 1A), where a common sensory pathway is wired to different target neurons in male and female animals. What is the genetic basis of this circuit switch? In *Drosophila*, an alternative splicing cascade converts sex chromosome status into sex-specific action of two terminal transcription factors, *fruitless* and *doublesex* (Billeter et al., 2006a). The action of *fruitless* is confined to the male nervous system, where the protein products of male-specific *fruitless* transcripts (collectively termed Fru^{M}) present in about 2,000 neurons are critical for male behavior (Lee et al., 2000; Ito et al., 1996; Ryner et al., 1996). Numerous studies have shown that *fruitless* loss-of-function mutations can change the morphology of both central and sensory neurons (Kimura et al., 2005; Mellert et al., 2010) and the survival of central neurons (Kimura et al., 2005). However, in only one case has *fruitless* been shown to be necessary for a sexually dimorphic neuronal connection: *fruitless* is required

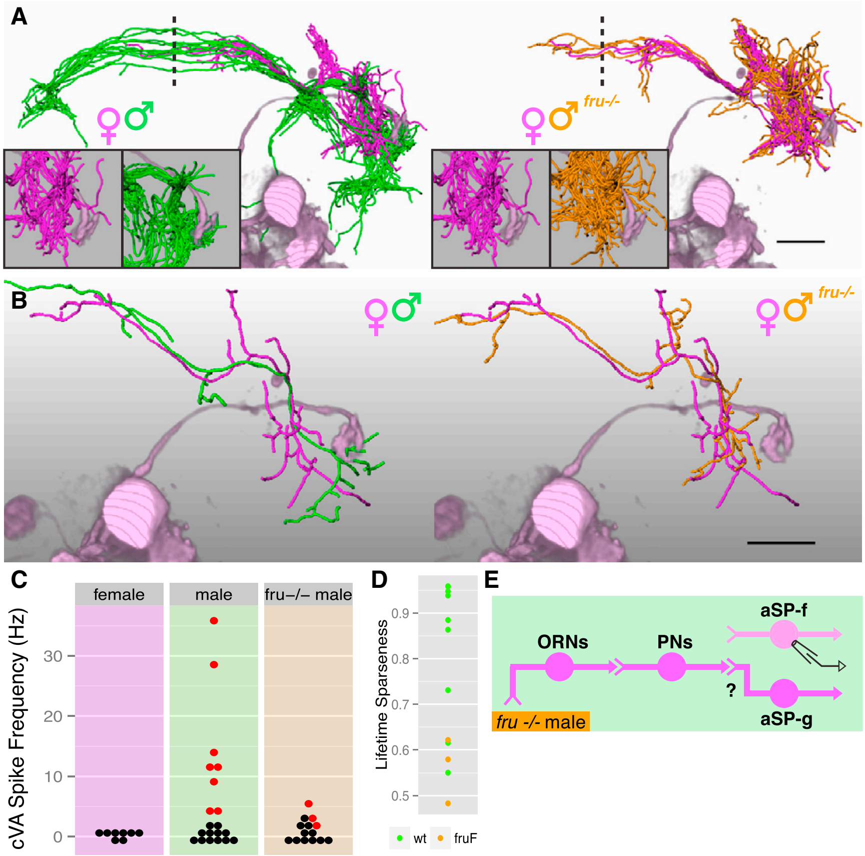

Figure 4. Fru^M Is Necessary for the Male Form of the Switch

(A and B) All (A) and single (B) dye-filled and reconstructed female, male, and $fru^{-/-}$ male *JK56* aSP-f neurons compared with volume-rendered DA1 PNs (pale magenta). Note that all male *JK56* aSP-f neurons are bilateral, whereas female and $fru^{-/-}$ male aSP-f neurons are largely unilateral. Dashed line marks midline. Insets in (A) show spatial relationship between LHN dendrites and DA1 PN axon terminals.

(C) Summary of cVA responses. Each dot is one neuron, significant cVA responses in red; nonsignificant responses are in black. See Table S1C for statistical analysis.

(D) Lifetime sparseness of wild-type male versus $fru^{-/-}$ male *JK56* aSP-f neurons (see the Experimental Procedures).

(E) Circuit model for $fru^{-/-}$ male brain. Note that we have not demonstrated a female-type connection from aSP-g dendrites to DA1 PN axons, so this is marked with a question mark.

Scale bars, 25 μm (A and B). See also Figure S4.

for survival of the Mind motor neuron in males, which in turn induces the formation of its target, the muscle of Lawrence (Nojima et al., 2010). We now show a direct effect of *fruitless* on brain wiring: a functionally validated change in connectivity between identified neurons.

First- (Manoli et al., 2005; Stockinger et al., 2005), second- (Stockinger et al., 2005; Datta et al., 2008), and third-order (Cachero et al., 2010; Yu et al., 2010; Ruta et al., 2010) olfactory neurons associated with pheromone signaling all express Fru^M protein in males but do not express *doublesex* (Rideout et al., 2010; Cachero et al., 2010). Does Fru^M therefore specify the male form of the bidirectional circuit switch? We used a heteroallelic loss-of-function combination fru^F/ fru^{4-40} (henceforth $fru^{-/-}$; Experimental Procedures) to remove Fru^M from all *fruitless*-expressing neurons (Figure S4A) and examined the morphology of the 6–7 aSP-f neurons labeled by the sparse *JK56* driver line (Figure S1A) in wild-type males, females, and $fru^{-/-}$ males.

In $fru^{-/-}$ males the ventral lateral horn lacked male-specific aSP-f dendrites (Figure S4B), closely resembling the female pattern. We performed whole-cell recordings to examine whether single aSP-f neurons were morphologically and functionally feminized in these $fru^{-/-}$ males. Wild-type female *JK56* aSP-f neurons showed no dendritic overlap with DA1 PNs and had unilateral axonal projections, whereas wild-type male aSP-f neurons contacted DA1 PNs and had bilateral axonal projections (Figure 4A). $fru^{-/-}$ neurons had minimal overlap with DA1 PNs and no contralateral projections (Figure 4A) and therefore resembled wild-type female neurons. Morphological clustering confirmed this impression (Figure S4E). Most (10/14) reconstructed $fru^{-/-}$ neurons coclustered with female neurons, with the remaining neurons displaying unusual projections in the dorsal lateral horn, which is never innervated by wild-type neurons of either sex. cVA elicited very weak spiking responses (range 2–6 Hz) in 3/14 $fru^{-/-}$ aSP-f neurons, two of which had aberrant morphology. In contrast 8/20 neurons in control males showed responses (range 4–36 Hz) (Figure 4C). This difference in response magnitude was statistically significant (Table S1C). The few odor-responsive $fru^{-/-}$ aSP-f neurons were also more broadly tuned than were their wild-type male counterparts (Figure 4D; Table S1C). Thus, although this $fru^{-/-}$ heteroallelic combination does not result in feminization of all neurons, Fru^M is clearly necessary to establish the male form of the circuit switch (Figure 4E). This parallels the observation that the same *fruitless* loss-of-function combination disrupts normal male courtship but does not lead to full behavioral feminization (Demir and Dickson, 2005).

Fru^M Specifies the Male Form of the Circuit Switch

Many studies have demonstrated that *fruitless* mutations selectively disrupt male behavior, leading to the influential hypothesis that Fru^M builds the potential for male sexual behavior into the fly nervous system (Baker et al., 2001). This hypothesis was dramatically validated by studies in which misexpression of Fru^M in females was sufficient to recapitulate many steps of male courtship behavior (Manoli et al., 2005; Demir and Dickson, 2005) or to produce masculinized courtship song (Clyne and Miesenböck, 2008). If Fru^M can specify male behavior, can it also specify the male form of the circuit switch in higher olfactory neurons?

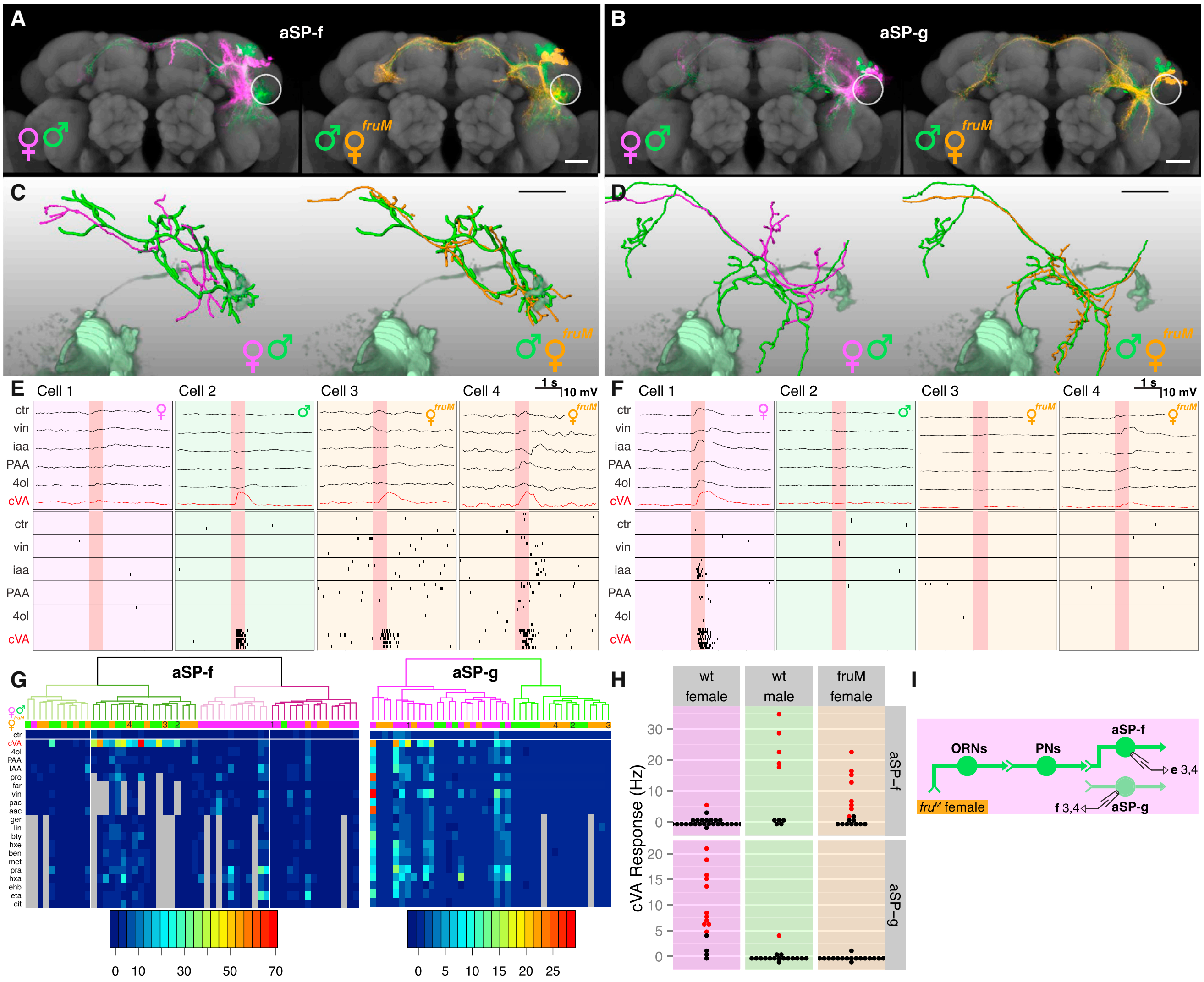

Figure 5. Fru^M Specifies the Male Form of the Circuit Switch

(A and B) Z projections of female, male, and *fru^M* mutant female neuroblast clones on a reference brain; the ventral lateral horn is marked with a white circle.

(C and D) Single dye-filled and reconstructed female, male, and *fru^M* female (C) aSP-f and (D) aSP-g neurons compared with volume-rendered DA1 PNs (pale green).

(E and F) Physiological data for aSP-f and aSP-g LHNs. These two panels are arranged in a 2-row × 4-column grid. The top row shows averaged current clamp recordings of each LHN shown in (C) and (D) (cells 1–3) and one additional mutant neuron. Row 2 shows raster plots for the same neurons.

(G) Mean odor responses of aSP-f (left) and aSP-g (right) neurons displayed as heatmap. Columns (i.e., neurons) are ordered by a dendrogram of morphological similarity between each neuron at the top of the panel. Dendrograms are split into colored subclusters. Below each dendrogram, one row indicates the sex of each neuron. Neurons displayed in (E) and (F) (cells 1–4) are highlighted with numbers (1–4) in the first row. Summary physiological data are presented in subsequent rows; each column is one neuron, and each row represents an odorant. Each box represents the color-coded average spiking frequency of a median of six odor trials. Gray boxes indicate odorants not tested.

(H) Summary of cVA responses. Each dot is one neuron, red for significant cVA response; nonsignificant responses are in black (8/18 aSP-f neurons and 0/17 aSP-g neurons responsive in *fru^M* females). See Table S1 for statistical analysis. All these neurons are labeled by the *JK1029* driver.

(I) Circuit model for *fru^M* female brain. Labels refer to cells in (E) or (F).

Scale bars, 25 μm. Pale red bars in (E) and (F) mark 500 ms odor presentation. See also Figure S5.

We used mosaic analysis with a repressible cell marker (MARCM) (Lee and Luo, 1999) to label *fru*+ neuronal clusters in females expressing Fru^M in all *fru*+ neurons (*fru^M* females [Demir and Dickson, 2005]). Examining MARCM clones labeling 16 sexually dimorphic clusters (Cachero et al., 2010), we found that nine clusters were indistinguishable from wild-type males, both in morphology and cell number; four clusters were not transformed (Ostrovsky, 2011). Completely masculinized clones included aSP-f (Figure 5A) and aSP-g (Figure 5B), components of the circuit switch. We recorded and filled LHNs in *fru^M* females and found that single reconstructed neurons were morphologically indistinguishable from male neurons (Figures 5C, 5D, and

5G; Figure S5A). cVA elicited spiking responses in about half of *fru*^{M} female aSP-f neurons (Figures 5E, 5G, and 5H), whereas no *fru*^{M} aSP-g neurons showed cVA spiking responses (Figures 5F–5H; Table S1). Fru^{M} therefore specifies the male form of the circuit switch, coordinating both the connection of aSP-f neurons to, and the disconnection of aSP-g neurons from, pheromone input (Figure 5I).

Selectively Masculinizing *fru*+ LHNs Can Flip the Switch

The experiments in Figures 4 and 5 demonstrate that Fru^{M} is both necessary and sufficient for the male form of the bidirectional circuit switch. Fru^{M} is expressed in <5% of the neurons in the fly brain, but these include pheromone-responsive first-, second-, and third-order olfactory neurons. We therefore asked whether selectively masculinizing *fru*+ LHNs in an otherwise female brain is sufficient to transform these neurons. We used null mutants in the *transformer (tra)* gene: *tra*^{1} mutant females are morphologically and behaviorally completely masculinized and loss of *tra* can masculinize individual somatic cells in a cell-autonomous manner (Baker and Ridge, 1980). Because *fru*+ LHNs do not express *doublesex*, any transformation should depend on Fru^{M}.

We generated MARCM clones homozygous mutant for *tra*^{1}, masculinizing these neurons in female brains (Kimura et al., 2008). aSP-f and aSP-g (Figures 6A and 6B) *tra*^{1} clones were morphologically indistinguishable from wild-type male clones, even when aSP-f or aSP-g were the only *tra*-deficient *fru*+ clones in the brain. This indicates a cell-autonomous effect. *tra*^{1} aSP-h neurons were also masculinized (Figures S5D and S5E).

In order to determine whether this morphological transformation was reflected functionally, we performed whole-cell recordings in females containing labeled (and therefore masculinized) LHN clusters (Figure S6; Experimental Procedures). Because of the stochastic nature of MARCM, each animal was examined on the electrophysiology rig to determine if GFP-labeled mutant clones were present in the lateral horn (n = 297 flies). In total we observed 15 aSP-f, 9 aSP-g, and 17 aSP-h clones, from which we recorded 14, 9, and 8 single neurons, respectively. Morphologically, individual *tra*^{1} aSP-f and aSP-g (Figures 6C and 6D) neurons were completely masculinized, coclustering with their wild-type male counterparts (Figure 6G).

Transformed aSP-g neurons had dendrites outside the ventral lateral horn, so we strongly predicted that they would lose their cVA responses. Indeed, no *tra*^{1} aSP-g neurons responded to cVA (Figures 6F–6H). Conversely, transformed aSP-f neurons have dendrites close to the axon terminals of female DA1 PNs. Is this sufficient for them to form functional connections? Strikingly, we observed cVA spiking responses in 5/14 *tra*^{1} mutant aSP-f neurons (Figures 6E, 6G, and 6H). *tra*^{1} mutant aSP-h neurons also gained male-type responses (Figures S5F and S5G; see Table S1C for full statistical tests). We conclude that the cell-autonomous transformation of *fru*+ LHNs is sufficient to recapitulate male form and function (Figure 6I).

DISCUSSION

Our study reveals principles of neural circuit organization and development that are of general significance. First, we show that two populations of neurons, present in both sexes, show reciprocal, sex-specific responses to the same stimulus. Second, we demonstrate that these responses result from differential wiring of a common input to different outputs. Together, these results define an elegant principle of neural circuit organization: a developmental circuit switch directly analogous to an electrical changeover (or single pole, double throw, SPDT) switch that efficiently reroutes a common input signal to one of two possible outputs. This model appears directly applicable to sex-specific processing of mouse pheromones, including ESP1 and Darcin (Haga et al., 2010; Stowers and Logan, 2010), but not to *Caenorhabditis elegans* ascarosides, where recent data suggest wiring differences may not be required (Jang et al., 2012; White and Jorgensen, 2012). The electrical changeover switch is the prototype for a wide-range of electrical switches in which concerted changes involving three or more contacts reroute signals (Horowitz and Hill, 1989); it is very likely that neural circuits, including those involved in pheromone processing, contain more complex switches or assemblies of multiple switches that elaborate on the basic mechanism that we have described here. Indeed, we previously identified over 700 sites of dimorphic neuronal overlap that may form such switches in other sensory pathways, multimodal interneurons, or motor circuits across the fly brain (Cachero et al., 2010).

Third, we identify sex-specific placement of target neuron dendrites as the primary cellular basis of the switch that we have described. This contrasts with earlier studies of this circuit that proposed that axonal dimorphism (Datta et al., 2008) or neurons present only in one sex (Ruta et al., 2010) were the key dimorphic element. Regarding axonal dimorphism, Datta et al. (2008) hypothesized that a male-specific extension of DA1 PN axon terminals is the basis of differential wiring in this system, and Ruta et al. (2010) subsequently proposed that this extension synapses with the dendrites of aSP-f LHNs in males. The large shifts in dendritic position that we observe in aSP-f and aSP-g neurons mean the male-specific extension of DA1 PNs cannot be sufficient for rewiring. Is it necessary? In our mosaic masculinization experiments, aSP-f and aSP-h neurons adopt male morphology and pheromone responses in a brain in which other neurons (including DA1 PNs) are female. Therefore, the male-specific ventral extension is either not necessary for differential wiring or is a secondary consequence of changes in the dendrites of post-synaptic LHNs. Of course, this extension may increase contact between DA1 PNs and aSP-f and aSP-h LHNs, strengthening responses of those LHNs in males. All three mechanisms (dendritic and axonal dimorphisms, dimorphic cell numbers) are likely relevant to different degrees in different circuits.

Fourth, having defined this bidirectional switch, we demonstrate that its male form is specified by the *fruitless* gene. We show that this transcription factor has a dual function, coordinating the disconnection of one group of target neurons and the connection of the other. Fifth, we show that masculinization of third-order neurons alone is sufficient for functional rewiring. Although previous studies have demonstrated a cell-autonomous effect of *fruitless* on neuronal morphology (Kimura et al., 2005, 2008; Ito et al., 2012), we now demonstrate a difference in functional connectivity. This is surprising because many would

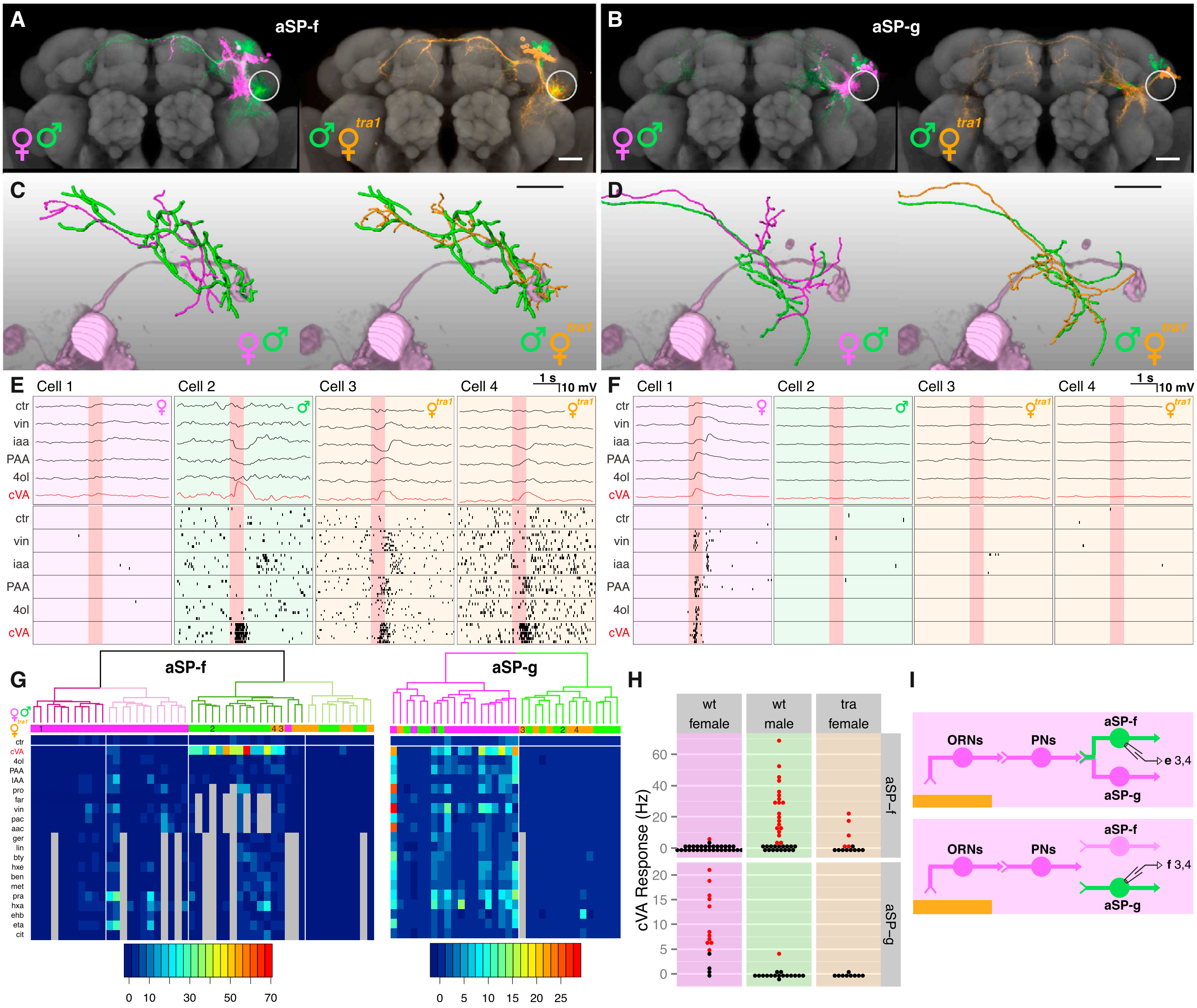

Figure 6. Selective Masculinization of *fru*+ LHNs Can Flip the Circuit Switch

(A and B) Z projections of female, male, and *tra*[1] mutant female neuroblast clones on a reference brain; the ventral lateral horn is marked with a white circle. *tra*[1]-transformed (A) aSP-f and (B) aSP-g clones in mosaic females are indistinguishable from their male counterparts.

(C and D) Single-filled and reconstructed female, male, and *tra*[1] female (C) aSP-f and (D) aSP-g neurons compared with volume-rendered DA1 PNs (pale magenta).

(E and F) Physiological data for (E) aSP-f and (F) aSP-g LHNs. These two panels are arranged in a 2-row × 4-column grid. The top row shows averaged current clamp recordings of each LHN from (C) and (D) (cells 1–3) and one additional mutant neuron. Row 2 shows raster plots for the same neurons.

(G) Mean odor responses of aSP-f (left) and aSP-g (right) neurons displayed as heatmap. Columns (i.e., neurons) are ordered by a dendrogram of morphological similarity between each neuron at the top of the panel. Dendrograms are split into colored subclusters. Below each dendrogram, one row indicates the sex of each neuron. Neurons displayed in (E) and (F) (cells 1–4) are highlighted with numbers (1–4) in the first row. Summary physiological data are presented in subsequent rows; each column is one neuron, and each row represents an odorant. Each box represents the color-coded average spiking frequency of a median of six odor trials. Gray boxes indicate untested odorants.

(H) Responses of both aSP-f (top) and aSP-g (bottom) neurons in *tra*[1] females are significantly different from wild-type females (see Table S1C for statistical analysis).

(I) Circuit models for female brains containing either a transformed *tra*[1] aSP-f (top) or *tra*[1] aSP-g (bottom) clone. Labels refer to cells in (E) or (F).

Scale bars, 25 µm. Pale red bars in (E) and (F) mark 500 ms odor presentation.

See also Figure S6.

predict that connectivity changes would depend on coordinate regulation of genes in synaptic partner neurons. Such simplicity has evolutionary implications: it may allow variation in circuit structure and ultimately in behavior, through evolution of *cis*-regulatory elements, as previously shown for somatic characters, such as wing spots (Prud'homme et al., 2007).

Sixth, studies of pheromone processing in general and cVA processing in particular have emphasized a labeled line processing model. However, our data indicate that both narrowly (aSP-f) and broadly tuned (aSP-h) cVA-responsive neurons coexist in males. Likewise in females, aSP-g neurons respond to cVA and general odors, such as vinegar, but only cVA responses depend on the Or67d receptor. It will be very interesting to determine the circuit origin and behavioral significance of this integration of odor channels. For example, it seems reasonable to speculate that coincidence of cVA and food odors could interact in a supralinear way to promote female courtship or egg laying. This parallels the convergence in the lateral horn of a labeled line responsive to non-cVA fly odors (Or47b/VA1lm neurons) and one responsive to a specific food odorant, phenylacetic acid, that acts as a male aphrodisiac (Grosjean et al., 2011).

Our study naturally raises additional questions. The action of *fruitless* within fewer than 5% of the neurons in the fly brain can specify behavior (Demir and Dickson, 2005; Manoli et al., 2005), and we now show that it can reroute pheromone signals within those neurons. But what is the behavioral relevance of this particular bidirectional switch? Testing this will require the development of sensitive behavioral assays of cVA processing and a reliable genetic approach to control this switch without affecting the many other dimorphic elements in sensory and motor circuits (Kimura et al., 2005, 2008; Clyne and Miesenböck, 2008; Cachero et al., 2010; Kohatsu et al., 2011; von Philipsborn et al., 2011). Indeed, it remains to be seen whether flipping a single switch in sensory processing is sufficient to engage motor behavior typical of the opposite sex without masculinizing downstream circuitry. We note that Clyne and Miesenböck (2008) could force the production of courtship song by activating *fruitless*-positive neurons in headless females but were almost never successful in intact females.

Another open question concerns the functional significance of female aSP-f and male aSP-g neurons, which do not respond to cVA or other tested odors. Do they receive input at all? One possibility, based on our in silico analysis of the brain-wide 3D maps in Chiang et al. (2011), is that they receive gustatory input, perhaps from contact pheromones, although further work is necessary to test this hypothesis. Finally, which genes does *fruitless* regulate in order to differentially wire the switch? Our clonal transformation experiments strongly support our earlier proposal (Cachero et al., 2010) that male and female aSP-f/g/h clusters are generated by neuroblasts common to both sexes but that those neurons develop in a sex-specific manner. Therefore, cell-surface molecules required for dendritic guidance are plausible targets. It will be intriguing to see if the same *fru*-dependent factor(s) direct(s) male aSP-f and female aSP-g dendrites to the ventral lateral horn and, more generally, whether *fruitless* acts on conserved downstream targets across all the dimorphic neurons in the fly brain (Cachero et al., 2010; Ito et al., 2012).

EXPERIMENTAL PROCEDURES

Fly Stocks

The *fruitless*^{Gal4} (*fru*^{Gal4}), *fru*^{F}, *fru*^{M}, *tra*^{1}, and *Or67d*^{Gal4} stocks were as described previously (Kimura et al., 2008; Kurtovic et al., 2007; Demir and Dickson, 2005; Baker and Ridge, 1980) (see Extended Experimental Procedures). *JK56* and *JK1029* are Split Gal4 enhancer trap P-element insertions of the Herpes Simplex VP16 activation domain (*VP16-AD*) (Luan et al., 2006), which were identified in a screen of 2,000 new insertions generated by our group (Extended Experimental Procedures). MARCM labeling of *tra*^{1} mutant clones used *y w hs-FLP UAS-mCD8-GFP / + ; UAS-mCD8-GFP FRT*^{G13} */ + ; tra*^{1} *FRT*^{2A} *fru*^{Gal4} */ tubP-Gal80 FRT*^{2A} flies. For anatomical experiments, MARCM clones were generated by heat shock of first-instar larvae for 17 min (males) or 23 min (females) at 37°C 0 hr–3 hr after larval hatching. For whole-cell recordings of *tra*^{1} mutant clones, heatshock time was extended to 1.5 hr (males and females), increasing clone frequency.

Immunochemistry

Immunochemistry was as described previously (Jefferis et al., 2007), except that blocking was overnight at 4°C. For Fru^{M} staining, fixation was in 2% PFA for 30 min on ice. Primary antibodies included mouse anti-nc82 (Wagh et al., 2006) (DSHB, University of Iowa) 1:20–1:40, chicken anti-GFP (Abcam, ab13970), and rabbit anti-Fru^{M} (rabbit polyclonal against male-specific 101 amino acids of Fru^{M} [Billeter et al., 2006b], gift of S. Goodwin) 1:400. Secondary antibodies (all from Life Technologies) included Alexa-568 anti-mouse (A-11004) 1:1,200, Alexa-633 anti-mouse (A-21052) 1:1,200, Alexa-488 anti-chicken (A-11039) 1:1,200, and Alexa-568 anti-rabbit (A-11011) 1:1,200. Filled neurons were visualized with Streptavidin Alexa-568 (S-11226) 1:1,300.

Image Acquisition and Analysis

Confocal stacks were acquired on a Zeiss 710 with a 40× NA1.3 oil objective, voxel resolution 0.46 × 0.46 × 1 μm. Images were registered to the IS2 template brain (Cachero et al., 2010) with the Computational Morphometry Toolkit (CMTK, http://www.nitrc.org/projects/cmtk). Neuron tracing used the skeletonize module (Evers et al., 2005) in Amira (VSG). Tracings were transformed to the left brain hemisphere using the AnalysisSuite package (https://github.com/jefferis/AnalysisSuite) written in R (http://www.r-project.org). Amira was used for 3D visualization. See http://jefferislab.org/si/frulhns for details and data download. Morphological analysis of traced neurons in R used an algorithm that scores the similarity of the local geometry of two neurons by calculating the distance between matching points and the dot products of the tangent vectors (see Extended Experimental Procedures for details and links to R code).

Electrophysiology

Recordings were made from 2- to 3-day-old flies essentially as described previously (Wilson et al., 2004), with the changes indicated in the Extended Experimental Procedures. A different protocol was developed for recording mutant clones in *tra*^{1} MARCM females (Extended Experimental Procedures). Single glomerulus stimulation was performed largely as described by Ruta et al. (2010), with modifications indicated in the Extended Experimental Procedures. Field recordings were performed to ensure that animals were odor-responsive (Extended Experimental Procedures). Data acquisition and initial analysis were carried out in Igor Pro with the NeuroMatic analysis software package (Jason Rothman, University College London; see http://neuromatic.thinkrandom.com); subsequent analysis was in R (Extended Experimental Procedures).

We quantified odor responses by finding the mean spike number in 500 ms window starting 150 ms after valve opening, subtracting the mean spike number for control stimulus. We assessed significance by an exact one-sided Poisson test of the number of spikes to odor and control stimuli using data from four trials per cell. We adjusted raw p values to control the false discovery rate (Benjamini and Hochberg, 1995) using R's p.adjust function; cells were declared significant for FDR adjusted $p < 0.01$.

Odor Stimulation

Odorant delivery used a custom odor delivery device (ODD; Extended Experimental Procedures and http://jefferislab.org/si/odd). All odorants were of the highest purity available and were prepared 1:100 v/v in mineral oil (Sigma, M8410), except propionic, butyric, and acetic acid, which were dissolved 1:100 v/v in water, and phenylacetic acid, which was diluted 1:200 w/v in water. cVA was undiluted.

Odorant abbreviations include ctr, mineral oil control; cVA, 11-*cis*-vaccenyl acetate; 4ol, butanol; PAA, phenylacetic aldehyde; IAA, isoamyl acetate; pro, propionic acid; far, farnesol; vin, apple cider vinegar; pac, phenylacetic acid;

aac, acetic acid; ger, geranyl acetate; lin, linalool; bty, butyric acid; hxe, E2-hexenal; ben, benzaldehyde; met, methyl salicylate; pra, propyl acetate; hxa, 1-hexanol; ehb, ethyl 3-hydroxybutyrate; eta, ethyl acetate; cit, b-citronellol. (See http://jefferislab.org/si/frulhns for detailed odorant descriptions.)

SUPPLEMENTAL INFORMATION

Supplemental Information includes Extended Experimental Procedures, seven figures, and one table and can be found with this article online at http://dx.doi.org/10.1016/j.cell.2013.11.025.

ACKNOWLEDGMENTS

We gratefully acknowledge initial tuition in fly electrophysiology from Glenn Turner in the laboratory of Gilles Laurent, while G.S.X.E.J. was a Wellcome Trust Advanced Training Fellow. We also thank Glenn Turner and Rachel Wilson for subsequent advice. We thank P. Hasel, B. Gyenes, G. Johnson, and J. Roote for assistance with Split Gal4 screening, A. Hodge and the LMB workshops for the odor delivery device, and R. Benton, B.J. Dickson, S. Goodwin, M. Landgraf, the Bloomington Stock Center, and the Developmental Studies Hybridoma Bank for fly stocks and antibodies. We thank R. Benton, J.C. Billeter, D. Logan, L. Luo, L. Newman, A.L. Taylor Tavares, G. Turner, and members of the Jefferis group for comments on the manuscript and R. Wilson, K. Scott, and M. Chatzigeorgiou for discussions. This study made use of the Computational Morphometry Toolkit, supported by the National Institute of Biomedical Imaging and Bioengineering. This work was supported by the Medical Research Council (MRC file reference MC_U105188491) and the European Research Council (to G.S.X.E.J.), by LMB Graduate Scholarships (to J.K. and A.O.), and by an EMBO long-term fellowship (to S.F.).

Received: August 19, 2013
Revised: October 2, 2013
Accepted: November 8, 2013
Published: December 19, 2013

REFERENCES

Baker, B.S., and Ridge, K.A. (1980). Sex and the single cell. I. On the action of major loci affecting sex determination in Drosophila melanogaster. Genetics *94*, 383–423.

Baker, B.S., Taylor, B.J., and Hall, J.C. (2001). Are complex behaviors specified by dedicated regulatory genes? Reasoning from Drosophila. Cell *105*, 13–24.

Benjamini, Y., and Hochberg, Y. (1995). Controlling the false discovery rate: a practical and powerful approach to multiple testing. J. R. Stat. Soc. Ser. B Stat. Methodol. *57*, 289–300.

Billeter, J.C., Rideout, E.J., Dornan, A.J., and Goodwin, S.F. (2006a). Control of male sexual behavior in Drosophila by the sex determination pathway. Curr. Biol. *16*, R766–R776.

Billeter, J.C., Villella, A., Allendorfer, J.B., Dornan, A.J., Richardson, M., Gailey, D.A., and Goodwin, S.F. (2006b). Isoform-specific control of male neuronal differentiation and behavior in Drosophila by the fruitless gene. Curr. Biol. *16*, 1063–1076.

Butler, B., Pirrotta, V., Irminger-Finger, I., and Nöthiger, R. (1986). The sex-determining gene tra of Drosophila: molecular cloning and transformation studies. EMBO J. *5*, 3607–3613.

Cachero, S., Ostrovsky, A.D., Yu, J.Y., Dickson, B.J., and Jefferis, G.S.X.E. (2010). Sexual dimorphism in the fly brain. Curr. Biol. *20*, 1589–1601.

Chiang, A.S., Lin, C.Y., Chuang, C.C., Chang, H.M., Hsieh, C.H., Yeh, C.W., Shih, C.T., Wu, J.J., Wang, G.T., Chen, Y.C., et al. (2011). Three-dimensional reconstruction of brain-wide wiring networks in Drosophila at single-cell resolution. Curr. Biol. *21*, 1–11.

Clyne, J.D., and Miesenböck, G. (2008). Sex-specific control and tuning of the pattern generator for courtship song in Drosophila. Cell *133*, 354–363.

Couto, A., Alenius, M., and Dickson, B.J. (2005). Molecular, anatomical, and functional organization of the Drosophila olfactory system. Curr. Biol. *15*, 1535–1547.

Datta, S.R., Vasconcelos, M.L., Ruta, V., Luo, S., Wong, A., Demir, E., Flores, J., Balonze, K., Dickson, B.J., and Axel, R. (2008). The Drosophila pheromone cVA activates a sexually dimorphic neural circuit. Nature *452*, 473–477.

Demir, E., and Dickson, B.J. (2005). fruitless splicing specifies male courtship behavior in Drosophila. Cell *121*, 785–794.

Dickson, B.J. (2008). Wired for sex: the neurobiology of Drosophila mating decisions. Science *322*, 904–909.

Dulac, C., and Kimchi, T. (2007). Neural mechanisms underlying sex-specific behaviors in vertebrates. Curr. Opin. Neurobiol. *17*, 675–683.

Ejima, A., Smith, B.P.C., Lucas, C., van der Goes van Naters, W., Miller, C.J., Carlson, J.R., Levine, J.D., and Griffith, L.C. (2007). Generalization of courtship learning in Drosophila is mediated by *cis*-vaccenyl acetate. Curr. Biol. *17*, 599–605.

Evers, J.F., Schmitt, S., Sibila, M., and Duch, C. (2005). Progress in functional neuroanatomy: precise automatic geometric reconstruction of neuronal morphology from confocal image stacks. J. Neurophysiol. *93*, 2331–2342.

Fisek, M., and Wilson, R.I. (2013). Stereotyped connectivity and computations in higher order olfactory neurons. Nat. Neurosci. Published online December 22, 2013. http://dx.doi.org/10.1038/nn.3613.

Fishilevich, E., and Vosshall, L.B. (2005). Genetic and functional subdivision of the Drosophila antennal lobe. Curr. Biol. *15*, 1548–1553.

Grosjean, Y., Rytz, R., Farine, J.P., Abuin, L., Cortot, J., Jefferis, G.S.X.E., and Benton, R. (2011). An olfactory receptor for food-derived odours promotes male courtship in Drosophila. Nature *478*, 236–240.

Ha, T.S., and Smith, D.P. (2006). A pheromone receptor mediates 11-*cis*-vaccenyl acetate-induced responses in Drosophila. J. Neurosci. *26*, 8727–8733.

Haga, S., Hattori, T., Sato, T., Sato, K., Matsuda, S., Kobayakawa, R., Sakano, H., Yoshihara, Y., Kikusui, T., and Touhara, K. (2010). The male mouse pheromone ESP1 enhances female sexual receptive behaviour through a specific vomeronasal receptor. Nature *466*, 118–122.

Horowitz, P., and Hill, W. (1989). The Art of Electronics, Second Edition (Cambridge: Cambridge University Press).

Hothorn, T., Hornik, K., Van De Wiel, M.A., and Zeileis, A. (2006). A lego system for conditional inference. Am. Stat. *60*, 257–263.

Ito, H., Fujitani, K., Usui, K., Shimizu-Nishikawa, K., Tanaka, S., and Yamamoto, D. (1996). Sexual orientation in Drosophila is altered by the satori mutation in the sex-determination gene fruitless that encodes a zinc finger protein with a BTB domain. Proc. Natl. Acad. Sci. USA *93*, 9687–9692.

Ito, H., Sato, K., Koganezawa, M., Ote, M., Matsumoto, K., Hama, C., and Yamamoto, D. (2012). Fruitless recruits two antagonistic chromatin factors to establish single-neuron sexual dimorphism. Cell *149*, 1327–1338.

Jang, H., Kim, K., Neal, S.J., Macosko, E., Kim, D., Butcher, R.A., Zeiger, D.M., Bargmann, C.I., and Sengupta, P. (2012). Neuromodulatory state and sex specify alternative behaviors through antagonistic synaptic pathways in C. elegans. Neuron *75*, 585–592.

Jefferis, G.S.X.E., Potter, C.J., Chan, A.M., Marin, E.C., Rohlfing, T., Maurer, C.R.J., Jr., and Luo, L. (2007). Comprehensive maps of Drosophila higher olfactory centers: spatially segregated fruit and pheromone representation. Cell *128*, 1187–1203.

Kazama, H., and Wilson, R.I. (2008). Homeostatic matching and nonlinear amplification at identified central synapses. Neuron *58*, 401–413.

Kimchi, T., Xu, J., and Dulac, C. (2007). A functional circuit underlying male sexual behaviour in the female mouse brain. Nature *448*, 1009–1014.

Kimura, K.I., Ote, M., Tazawa, T., and Yamamoto, D. (2005). Fruitless specifies sexually dimorphic neural circuitry in the Drosophila brain. Nature *438*, 229–233.

Kimura, K.I., Hachiya, T., Koganezawa, M., Tazawa, T., and Yamamoto, D. (2008). Fruitless and doublesex coordinate to generate male-specific neurons that can initiate courtship. Neuron *59*, 759–769.

Kohatsu, S., Koganezawa, M., and Yamamoto, D. (2011). Female contact activates male-specific interneurons that trigger stereotypic courtship behavior in Drosophila. Neuron *69*, 498–508.

Kurtovic, A., Widmer, A., and Dickson, B.J. (2007). A single class of olfactory neurons mediates behavioural responses to a Drosophila sex pheromone. Nature *446*, 542–546.

Lee, T., and Luo, L. (1999). Mosaic analysis with a repressible cell marker for studies of gene function in neuronal morphogenesis. Neuron *22*, 451–461.

Lee, G., Foss, M., Goodwin, S.F., Carlo, T., Taylor, B.J., and Hall, J.C. (2000). Spatial, temporal, and sexually dimorphic expression patterns of the fruitless gene in the Drosophila central nervous system. J. Neurobiol. *43*, 404–426.

Luan, H., Peabody, N.C., Vinson, C.R., and White, B.H. (2006). Refined spatial manipulation of neuronal function by combinatorial restriction of transgene expression. Neuron *52*, 425–436.

Manoli, D.S., Foss, M., Villella, A., Taylor, B.J., Hall, J.C., and Baker, B.S. (2005). Male-specific fruitless specifies the neural substrates of Drosophila courtship behaviour. Nature *436*, 395–400.

Masse, N.Y., Cachero, S., Ostrovsky, A.D., and Jefferis, G.S.X.E. (2012). A mutual information approach to automate identification of neuronal clusters in Drosophila brain images. Front. Neuroinform. *6*, 21.

Mellert, D.J., Knapp, J.M., Manoli, D.S., Meissner, G.W., and Baker, B.S. (2010). Midline crossing by gustatory receptor neuron axons is regulated by fruitless, doublesex and the Roundabout receptors. Development *137*, 323–332.

Nojima, T., Kimura, K.i., Koganezawa, M., and Yamamoto, D. (2010). Neuronal synaptic outputs determine the sexual fate of postsynaptic targets. Curr. Biol. *20*, 836–840.

Ostrovsky, A.D. (2011). A sexually dimorphic olfactory circuit in the fruit fly, Drosophila melanogaster. PhD thesis, University of Cambridge, Cambridge, UK.

Prud'homme, B., Gompel, N., and Carroll, S.B. (2007). Emerging principles of regulatory evolution. Proc. Natl. Acad. Sci. USA *104* (*Suppl 1*), 8605–8612.

Rideout, E.J., Dornan, A.J., Neville, M.C., Eadie, S., and Goodwin, S.F. (2010). Control of sexual differentiation and behavior by the doublesex gene in Drosophila melanogaster. Nat. Neurosci. *13*, 458–466.

Ruta, V., Datta, S.R., Vasconcelos, M.L., Freeland, J., Looger, L.L., and Axel, R. (2010). A dimorphic pheromone circuit in Drosophila from sensory input to descending output. Nature *468*, 686–690.

Ryner, L.C., Goodwin, S.F., Castrillon, D.H., Anand, A., Villella, A., Baker, B.S., Hall, J.C., Taylor, B.J., and Wasserman, S.A. (1996). Control of male sexual behavior and sexual orientation in Drosophila by the fruitless gene. Cell *87*, 1079–1089.

Schlief, M.L., and Wilson, R.I. (2007). Olfactory processing and behavior downstream from highly selective receptor neurons. Nat. Neurosci. *10*, 623–630.

Stockinger, P., Kvitsiani, D., Rotkopf, S., Tirián, L., and Dickson, B.J. (2005). Neural circuitry that governs Drosophila male courtship behavior. Cell *121*, 795–807.

Stowers, L., and Logan, D.W. (2010). Sexual dimorphism in olfactory signaling. Curr. Opin. Neurobiol. *20*, 770–775.

Tompkins, L., and Hall, J.C. (1983). Identification of Brain Sites Controlling Female Receptivity in Mosaics of DROSOPHILA MELANOGASTER. Genetics *103*, 179–195.

Touhara, K., and Vosshall, L.B. (2009). Sensing odorants and pheromones with chemosensory receptors. Annu. Rev. Physiol. *71*, 307–332.

van der Goes van Naters, W., and Carlson, J.R. (2007). Receptors and neurons for fly odors in *Drosophila*. Curr. Biol. *17*, 606–612.

von Philipsborn, A.C., Liu, T., Yu, J.Y., Masser, C., Bidaye, S.S., and Dickson, B.J. (2011). Neuronal control of Drosophila courtship song. Neuron *69*, 509–522.

Wagh, D.A., Rasse, T.M., Asan, E., Hofbauer, A., Schwenkert, I., Dürrbeck, H., Buchner, S., Dabauvalle, M.C., Schmidt, M., Qin, G., et al. (2006). Bruchpilot, a protein with homology to ELKS/CAST, is required for structural integrity and function of synaptic active zones in Drosophila. Neuron *49*, 833–844.

Wang, L., and Anderson, D.J. (2010). Identification of an aggression-promoting pheromone and its receptor neurons in Drosophila. Nature *463*, 227–231.

White, J.Q., and Jorgensen, E.M. (2012). Sensation in a single neuron pair represses male behavior in hermaphrodites. Neuron *75*, 593–600.

Willmore, B., and Tolhurst, D.J. (2001). Characterizing the sparseness of neural codes. Network *12*, 255–270.

Wilson, R.I., Turner, G.C., and Laurent, G. (2004). Transformation of olfactory representations in the Drosophila antennal lobe. Science *303*, 366–370.

Wu, M.V., and Shah, N.M. (2011). Control of masculinization of the brain and behavior. Curr. Opin. Neurobiol. *21*, 116–123.

Wyatt, T.D. (2003). Pheromones and Animal Behaviour: Communication by Smell and Taste (Cambridge: Cambridge University Press).

Yu, J.Y., Kanai, M.I., Demir, E., Jefferis, G.S.X.E., and Dickson, B.J. (2010). Cellular organization of the neural circuit that drives Drosophila courtship behavior. Curr. Biol. *20*, 1602–1614.

Supplemental Information

EXTENDED EXPERIMENTAL PROCEDURES

Additional data and computer code are available at http://jefferislab.org/si/frulhns. All 3D image data are available on a hard drive on request to GSXEJ.

Fly Stocks

The *fruitless*^{Gal4} (*fru*^{Gal4}) allele is a targeted insertion of the yeast transcription factor Gal4 into the P1 promoter of the *fruitless* gene (Demir and Dickson, 2005). In *fru*^{M} mutants, the entire 1,601 bp female-specific part of the S exon is deleted, enforcing male-specific splicing and thus expression of male-specific Fru^{M} isoforms (Demir and Dickson, 2005). In *fru*^{F} mutants, point mutations introduced at the male splice donor site of the S exon of *fruitless* abolish splicing at this site without altering the coding potential of the unspliced transcripts (Demir and Dickson, 2005). *fru*$^{4\text{-}40}$ is a lethal deletion in the *fruitless* locus, extending distally from *fru*^{4} for > 70 kb (Lee et al., 2000). Heteroallelic *fru*^{F} / *fru*$^{4\text{-}40}$ flies are referred to as *fru*$^{-/-}$ in this study. In *tra*^{1} loss-of-function mutants, the entire coding region of *transformer* is deleted (Butler et al., 1986). The *JK56* insertion was mapped by inverse PCR to cytological location 91B5, genomic coordinate 3R: 14410045(-), ~40 kb upstream of the *fruitless* locus. The *JK1029* insertion is located on 3R, close to the *fruitless* locus, but a genomic location is not yet available. Both *JK56* and *JK1029* were recombined with *Cha-Gal4-DBD*, an insertion that expresses a Gal4 DNA binding domain-leucine zipper fusion protein under control of the *Cha* promoter. *Or67d*^{Gal4} is a mutant knock-in allele in which the open reading frame of *Or67d* is replaced with that of the yeast transcriptional activator Gal4 (Kurtovic et al., 2007). Flies homozygous for *Or67d*^{Gal4} are referred to as *Or67d*$^{-/-}$ in this study.

Wild-type neuron clusters were targeted for whole-cell recordings in flies of the following genotypes:
UAS-mCD8-GFP / (+ or Y) ; UAS-mCD8-GFP / FRT^{G13} UAS-mCD8-GFP ; fru^{Gal4} / +
UAS-mCD8-GFP / (+ or Y) ; UAS-mCD8-GFP / + ; JK56-VP16AD Cha-Gal4-DBD / +
UAS-mCD8-GFP / (+ or Y) ; UAS-mCD8-GFP / + ; JK1029-VP16AD Cha-Gal4-DBD / +
Single glomerulus stimulation experiments were performed in flies of the following genotypes:
UAS-mCD8-GFP / (+ or Y); UAS-mCD8-GFP / Mz19-Gal4 UAS-mCD8-GFP; JK1029-VP16AD Cha-Gal4-DBD / +
UAS-mCD8-GFP / (+ or Y); UAS-mCD8-GFP / + ; JK1029-VP16AD Cha-Gal4-DBD Or67d^{Gal4} / +
Or67d mutant recordings were performed in flies of the genotype:
UAS-mCD8-GFP / (+ or Y); UAS-mCD8-GFP / + ; JK1029-VP16AD Cha-Gal4-DBD Or67d^{Gal4} / Or67d^{Gal4}
fru^{M} mutant clusters were targeted in female flies of the genotypes:
UAS-mCD8-GFP / + ; UAS-mCD8-GFP / + ; fru^{Gal4} / fru^{M}
UAS-mCD8-GFP / + ; UAS-mCD8-GFP / + ; JK1029-VP16AD Cha-Gal4-DBD / fru^{M}
fru^{F} mutant clusters were targeted in male flies (fru$^{-/-}$ males) of the genotype:
UAS-mCD8-GFP / Y ; UAS-mCD8-GFP / + ; fru$^{4\text{-}40}$ Cha-Gal4-DBD / fru^{F} JK56-VP16AD
tra^{1} mutant clones were targeted in female flies of the genotype:
y w hs-FLP UAS-mCD8-GFP / + ; UAS-mCD8-GFP FRT^{G13} / + ; tra^{1} FRT^{2A} fru^{Gal4} / tubP-Gal80 FRT^{2A}

MARCM

MARCM labeling of wild-type LHN clones used flies of the genotype:
y w hs-FLP UAS-mCD8-GFP / (+ or Y) ; UAS-mCD8-GFP FRT^{G13} / FRT^{G13} tubP-GAL80 ; fru^{Gal4} / +.
MARCM labeling of fru^{M} mutant clones used flies of the genotype:
y w hs-FLP UAS-mCD8-GFP / (+ or Y) ; UAS-mCD8-GFP FRT^{G13} / FRT^{G13} tubP-GAL80 ; fru^{Gal4} / fru^{M}

Immunohistochemistry

Prolonged incubation (2–3 days rotating at 4°C) with primary and secondary antibodies was required for homogeneous staining. Specimens were whole mounted in Vectashield (Vector Laboratories) on charged slides to avoid movement.

Image Acquisition and Analysis

Brains were imaged at 768 × 768 pixel resolution and 0.6 zoom factor. Images of dye-filled neurons were acquired with 2x (frame) averaging. Detail images were taken with a Plan-Apochromat 63x/1.4 Oil objective at 2–3x zoom and contained about 30 768 × 768 pixel slices with a voxel size of 0.06 × 0.06 × 0.15 μm. All images were taken using 16bit color depths.

For morphological analysis, the fully connected reconstruction was transformed into a "DotProperties" representation that retains only the 3D position of points along with the local heading (tangent vector) of the neurite (Masse et al., 2012). Neurons in this representation could then be compared in pairwise fashion. For each point on a neuron designated as the query neuron, the closest point on the target neuron was identified using a nearest neighbor algorithm (R package RANN, http://cran.r-project.org/web/packages/RANN). Each point pair was then given a score which was a function of the distance between them multiplied by the absolute dot product of the two tangent vectors. The similarity score for these two neurons was then simply the average of all the scores for all

the individual point pairs. The similarity score $S(Q,T)$ (and the corresponding distance score, $D(Q,T)$) for a query neuron, Q, and a target neuron, T, are precisely defined as follows:

$$S(Q,T) = \frac{1}{n}\sum_{i=1}^{n}\sqrt{|q_i \cdot t_j| e^{-\frac{d_{ij}^2}{2\sigma^2}}} \quad D(Q,T) = 1 - S(Q,T)$$

where n is the number of points in the query neuron, d_{ij} is the distance between point i in the query neuron and its nearest neighbor (point j) in the target neuron and q_i and t_j are the tangent vectors at these points. There is one free parameter, σ, which determines how close in space points must be to be considered similar; we set this value to 3 μm based on previous estimates of registration accuracy/biological variability in the fly brain (Jefferis et al., 2007; Yu et al., 2010). Similarity scores will range between 1 (identical) and 0 (completely different). This approach is implemented in R in the AnalysisSuite function WeightedNNBasedLinesetMatching (https://github.com/jefferis/AnalysisSuite/blob/master/R/Code/NeuroBlast/NeuriteMatchingFunctions.R#L164). For clustering, pairwise similarity scores were converted to distances by subtraction from 1 and clustering was performed using R function hclust using Ward's method.

Electrophysiology

Flies were reared on standard fly food at a 12-12 light-dark cycle at 25°C and 60% humidity. Fly preparations and recordings were essentially performed as described (Wilson et al., 2004), with the following changes. UV-curable glue (Kemxert, KOA-300) was used to fix the fly in custom holders which were photofabricated from a 0.025 mm thick sheet of 302 grade stainless steel (Photofabrication Limited). After the forelegs were removed, the proboscis was fixed to a piece of hair running perpendicular to the longitudinal axis of the body of the fly with wax. To prevent motion in the brain, muscle 16 (paired muscle dorsal to the esophagus) was removed with fine forceps. Gentle mechanical desheathing of the exposed brain was performed using fine forceps. Enzymatic desheathing was necessary in most cases to access cell bodies for whole-cell recordings. For this purpose, wide (~15 μm) desheathing pipettes were pulled from thin-filament (1.5 mm O.D. x 1.17 mm I.D.) borosilicate glass capillaries (Harvard Apparatus) and filled with a 0.22 μm syringe-filtered solution of 0.5 mg ml^{-1} collagenase type IV (Worthington or Abnova) in saline (see below). Gentle positive (~30 mm Hg) pressure was applied to rupture the glial sheath and then collagenase was allowed to diffuse into the tissue under reduced pressure (~10 mm Hg) for 1–2 min. Perfusion rate during experiments was 0.25 ml min^{-1}, controlled by a peristaltic pump. Saline was continuously bubbled with 95% O_2/ 5% CO_2 and frequently exchanged during the dissection and recording procedure. Only one neuron per brain was recorded and only neurons for which both physiological and morphological data (i.e., biocytin fill) could be obtained were included in this study (see Experimental Procedures; Tables S1A and S1B).

Because of the low probability of finding a transformed (i.e., GFP-positive) LHN clone in *tra*^{1} MARCM females, typically 10–20 animals had to be screened before the recording procedure could begin. Therefore, as soon as a fly was inserted into the holder, the next vial was put on ice in order to avoid unnecessary delays. Only 2–4 females were kept per vial to avoid repeated anesthesia over short periods of time. The proboscis was immobilized (see above) and tracheae were removed, but muscle 16 was left intact and no mechanical desheathing was initially performed. Flies were then transferred onto the microscope platform and only if labeled LHN clones were present in the right hemisphere the remaining steps were performed and recordings initiated. Recordings were only performed from neuroblast clones since reliable identification of single-cell clones was challenging.

Patch-clamp recordings were obtained using an Olympus BX51W1 upright microscope with IR-DIC illumination and a LUMPlanFL/IR 60x water immersion objective. A Grasshopper 143S camera (Point Grey Research) controlled by Micro-Manager software (http://www.micro-manager.org) was used to guide the recording electrode. Patch-clamp electrodes (7–9 MΩ) were pulled from thin-filament (1.5 mm O.D. x 1.17 mm I.D.) borosilicate glass capillaries (Harvard Apparatus) using a Zeitz DMZ Universal Puller (Zeitz - Instruments Vertriebs GmbH) and pressure-polished with a CPM-2 Coating and Polishing Microforge (ALA Scientific Instruments) mounted onto an Olympus CKX41 inverted microscope with a 50x LWD lens. Patch pipettes were filled with a solution of (in mM): Potassium Aspartate 125, $CaCl_2$ 0.1, HEPES 10, MgATP 4, NaGTP 0.5, EGTA 1.1, biocytin hydrazide (Life Technologies, B-1603) 13 (pH = 7.2, adjusted to 265 mOsm), that was filtered through a 0.22 μm syringe filter. Saline (external solution) was composed as follows (in mM): NaCl 103, KCl 3, NaH_2PO_4 1, $MgCl_2.6H_20$ 4, $CaCl_2.2H_20$ 1.5, $NaHCO_3$ 26, TES 5, glucose 10, trehalose 10 (pH = 7.25, adjusted to 275 mOsm). All chemicals were purchased from Sigma at the highest purity grade available, unless indicated otherwise. Signals were acquired on an Axoclamp 200B amplifier (Molecular Devices) via a CV-7B headstage and low-pass filtered at 10 kHz, digitized at 11.1kHz. Note that although the digitization frequency was below the Nyquist frequency for the amplifier's 10 kHz filter, we confirmed that any aliasing artifact in the 100-1000Hz range was not detectable (i.e., of much lower amplitude than other sources of noise) and had no impact on spike finding. Recording protocols were delivered via Igor Pro software (Wavemetrics) in conjunction with Nclamp (Jason Rothman, University College London, UK, see http://neuromatic.thinkrandom.com).

Field recordings were performed as follows: a patch pipette was filled with 0.22 μm syringe-filtered saline and lowered into the cell body layer dorsal of the mushroom body calyx under positive pressure (~10 mm Hg). Odor-evoked field responses were then recorded in current clamp mode. Field odor responses were typically visible as 0.1–0.3 mV downward potential deflections (Figure S2C).

Analysis

Initial analysis was carried out in Igor Pro using the NeuroMatic analysis software package (Jason Rothman, University College London, UK, see http://neuromatic.thinkrandom.com). Cells were accepted for analysis if spikes could be elicited by current injection and extracted using the following protocol. Current clamp traces were smoothed with a binomial algorithm at 180–550 Hz and action potentials ('spikes') were extracted by applying a negative threshold to the second derivative of the smoothed trace. The resultant spike times were then imported into the R statistical environment for further processing, statistical analysis and production of final graphics (our open source R code is available for download at http://jefferislab.org/si/frulhns). All quantities reported in the paper are mean ± s.d. unless otherwise indicated.

The odor tuning of responsive neurons was quantified using a measure of tuning curve sharpness (S, lifetime sparseness, Willmore and Tolhurst, 2001; Wilson et al., 2004). Lifetime sparseness ranges from 0 (for a neuron that responds equally to all odorants) to 1 (for a neuron that is excited by one odorant while showing no response to all others). In order to compute peak firing rates for aSP-f and aSP-g neurons, we calculated response rates in five 200 ms windows starting at 150 ms after valve opening, reporting the peak value for each cell.

When comparing odor responses between groups of cells we used a permutation approach implemented in the R library coin (Hothorn et al., 2006), specifically the oneway_test function. This is a nonparametric two-sample test analogous to a t test or Wilcoxon text to detect a shift between two populations, with the advantage over a Wilcoxon test that the magnitude (not just the rank) of responses is considered. The permutation approach means that there are no distributional assumptions (including equality of sample variances).

In order to assess the significance of subthreshold responses to cVA we first smoothed the membrane potential with a 45ms boxcar filter. We then calculated the peak response in a window from +200 to +800 ms after valve opening, subtracting a baseline value of the mean response between −1000 to 0 ms. We repeated the same procedure for a response window preceding odor presentation, generating a null distribution of pseudo responses from all of the traces available for each cell. The cVA responses were then compared with the pseudo responses using the non parametric oneway_test of the R coin package (Hothorn et al., 2006). Raw p values were adjusted to control the False Discovery Rate (Benjamini and Hochberg, 1995) using the p.adjust function in R; responses were declared significant for FDR adjusted $p < 0.01$.

Odor Stimulation

Odorants were kept in glass vials with glass wool (Acros Organics) to increase the effective surface area, thus facilitating odor headspace formation in the vial. A constant airstream of 2 l min^{-1} was directed at the fly throughout the recording. This airstream was composed of a carrier stream (1.75 l min^{-1}) and an odor stream (0.25 l min^{-1}) that was directed from a mineral oil control vial to an odor vial by a trigger-controlled solenoid valve. The rate between carrier and odor stream was set via two mass flow controllers (MFCs). The two streams were joined 70 cm from the end of the delivery tube, which measured 4 mm in diameter and was positioned 8 mm from the fly. All odor concentrations, as they leave the delivery tube, are therefore ~800x dilutions. Typical odor response latencies with this setup were 150–200 ms, as measured by spiking onset in our recordings and with a photoionization detector (mini-PID, Aurora Scientific) that was placed near the end of the delivery tube. In addition, we sometimes observed a quasi-instantaneous spiking onset in neurons (latency < 5 ms) that we ascribe to a change in pressure.

Several precautions were taken to account for false negative results, i.e., recordings from flies that were unable to respond to odors per se: (a) successful odor delivery was monitored with a photoionization detector in series with the animal (see above), (b) great care was taken during the preparation to fully expose the olfactory appendages of the fly without damaging them or the associated olfactory nerves, (c) in order to determine whether neurons were capable of generating action potentials ("spikes"), positive current was injected, and (d) neurons were inspected to establish if they exhibited at least one supra- or subthreshold response to odor. Recordings were only included if criteria (a–c) were fulfilled; if (d) was not fulfilled, a field recording (Figure S2C) was performed to ensure that the animal was odor-responsive (see above).

Single Glomerulus Stimulation

For single glomerulus stimulations, theta glass stimulation electrodes (Warner Instruments) were pulled with a 3–5 μm tip and filled with 2 mM acetylcholine chloride (Sigma) in saline. With the exception of the experiment in Figure S3D, both sides of the theta electrode were filled with identical solution (i.e., effectively a one-barreled electrode), using a separate bath electrode as ground. While there is therefore no liquid junction potential between the two barrels of the theta electrode, there is a liquid junction potential between the bath electrode (in saline) and the theta electrode (saline + acetylcholine). However, since the concentration of acetylcholine used in these experiments was very low (2 mM), this liquid junction potential is less than 0.2 mV.

Stimulation electrodes were inserted into the GFP-positive DA1 glomerulus. These recordings were performed in flies with an additional copy of *Mz19-Gal4* or $Or67d^{Gal4}$ in order to reliably identify the DA1 glomerulus. In order to reduce spontaneous activity in DA1 PNs, both antennae were removed with forceps. Square voltage pulses (500 ms), ranging from 0.2–10 V were delivered via the DAC output of the ITC-18 data acquisition interface (HEKA Elektronik) using a common bath electrode. This configuration results in both transient and sustained stimulation artifacts; the sustained artifacts (typically 1 mV/V stimulation) were fitted for each cell and subtracted before quantification. Stimulation depolarizes PNs both chemically and electrically (see Figure S3D). For control stimulations the stimulation electrode was moved to neighboring glomeruli in the dorsal antennal lobe. Note that although this controls for stimulation spreading 'across' glomeruli, it does not rule out input from more ventral glomeruli to (broadly odor-tuned) female aSP-g

neurons. After control stimulations of non-DA1 glomeruli, the stimulation electrode was repositioned in DA1 to ensure that there was no drop in responsivity throughout the recording procedure. A small number of experiments investigated the minimum stimulation voltage required to elicit a postsynaptic response, using stimulation intensities as low as 10 mV.

To measure stimulation latency we first applied a digital boxcar filter at 2.2 kHz to the signals. We normalized the voltage level by subtracting the mean value between 0 and 0.5 ms after stimulation. Responses to both DA1 and control glomerulus stimulation were plotted (Figure S3B) and binned in 0.5 ms intervals. We then tested for divergence between voltage traces for DA1 and control glomerulus stimulation by carrying out a Wilcoxon rank sum test within each 0.5 ms bin. The latency was then defined as the time after which the DA1 trace remained significantly larger ($p < 0.05$, one-tailed) than the control trace for the remainder of the stimulation period.

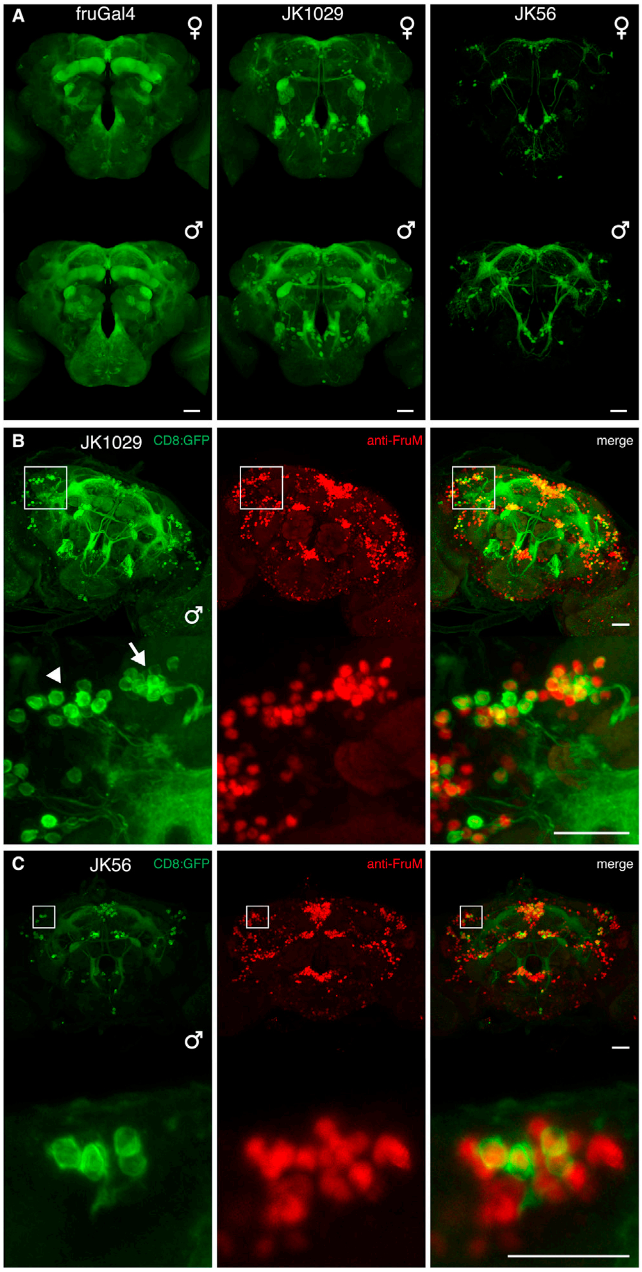

Figure S1. *JK1029* and *JK56* Label Subsets of *fru*+ Neurons, Related to Figure 1

(A) Expression patterns of *fru*^{Gal4}, *JK1029* and *JK56* when driving membrane-targeted GFP. *fru*^{Gal4} labels all *fru*+ neurons but expression is sparser in *JK1029* and *JK56* which label ~60% and ~10% of *fru*+ neurons in the central brain, respectively, when crossed to *Cha-Gal4-DBD*. In general, expression is dimorphic for all three driver lines, with more neurons being labeled in males. However this is not true for all neuronal clusters labeled by these lines.

(B and C) *JK1029* and *JK56* label perfect subsets of *fru*+ neurons in the central brain as demonstrated by immunostainings against Fru^{M}. Boxed regions in top panels in (B) and (C) are magnified in the bottom panels. Expression patterns of *JK1029* (B, top left) and *JK56* (C, top left) driving membrane-targeted GFP. All GFP-positive neurons also express Fru^{M}. (B) *JK1029* labels subsets of aSP-f (arrow) and aSP-g (arrowhead) as visible in Fru^{M} immunostainings. (C) *JK56* labels even smaller subsets of aSP-f (boxed region) and aSP-g (not shown), as visible in Fru^{M} immunostainings. *JK1029* and *JK56* hemi-drivers were crossed to *Cha-Gal4-DBD*. All scale bars 25 μm.

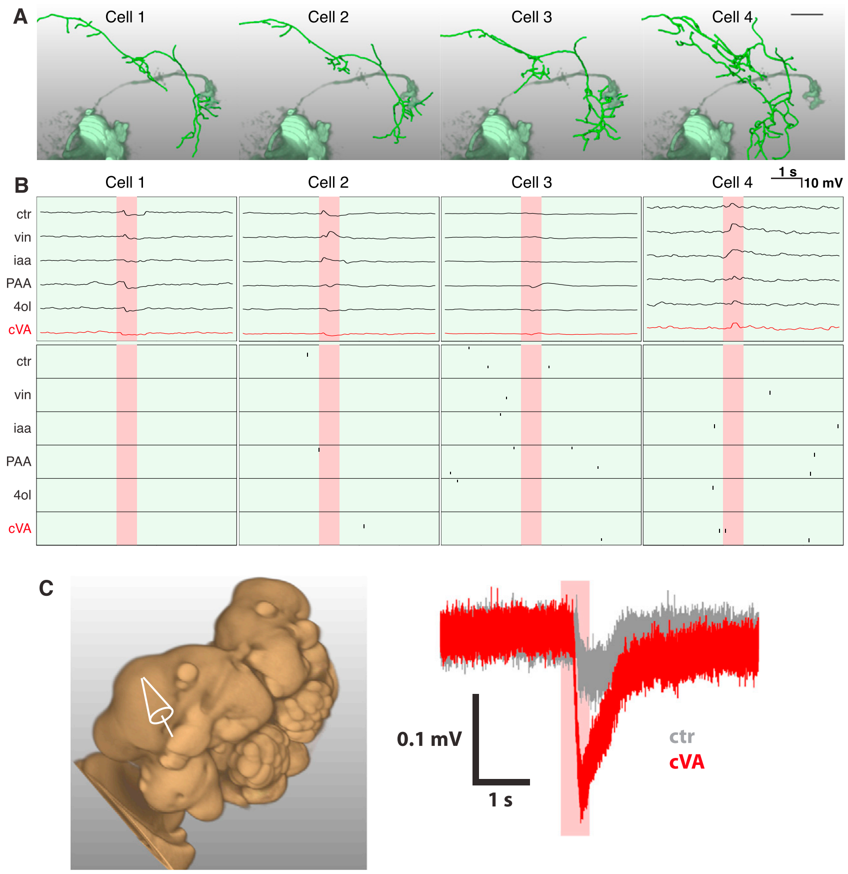

Figure S2. Examples of Silent Wild-Type Male aSP-f Neurons, Related to Figure 2

(A) Single dye-filled and reconstructed wild-type male aSP-f neurons (green) compared with volume rendered DA1 PNs (pale green).

(B) Physiological data for the neurons displayed in (A). The top row shows averaged current clamp recordings of each neuron, the bottom row shows raster plots for the same neurons. Even though their dendrites overlap with DA1 PNs, Cells 1–3 do not show excitatory responses. Instead, Cells 1–2 are inhibited by cVA and other odorants. Cell 4 is a morphological exception, with dendrites outside the ventral lateral horn (in contrast to 36/37 male aSP-f neurons).

(C) Extracellular field recordings were performed after recordings of non odor-responsive neurons (see the Experimental Procedures). Pipette marks recording site anterior-dorsal of Kenyon cell layer (left). Downward deflections in extracellular potential elicited by presentation of cVA (right). Note that the mineral oil control (ctr) elicits a small response. Scale bar in (A) 25 μm. Pale red bars in (B) and (C) mark 500 ms odor presentation.

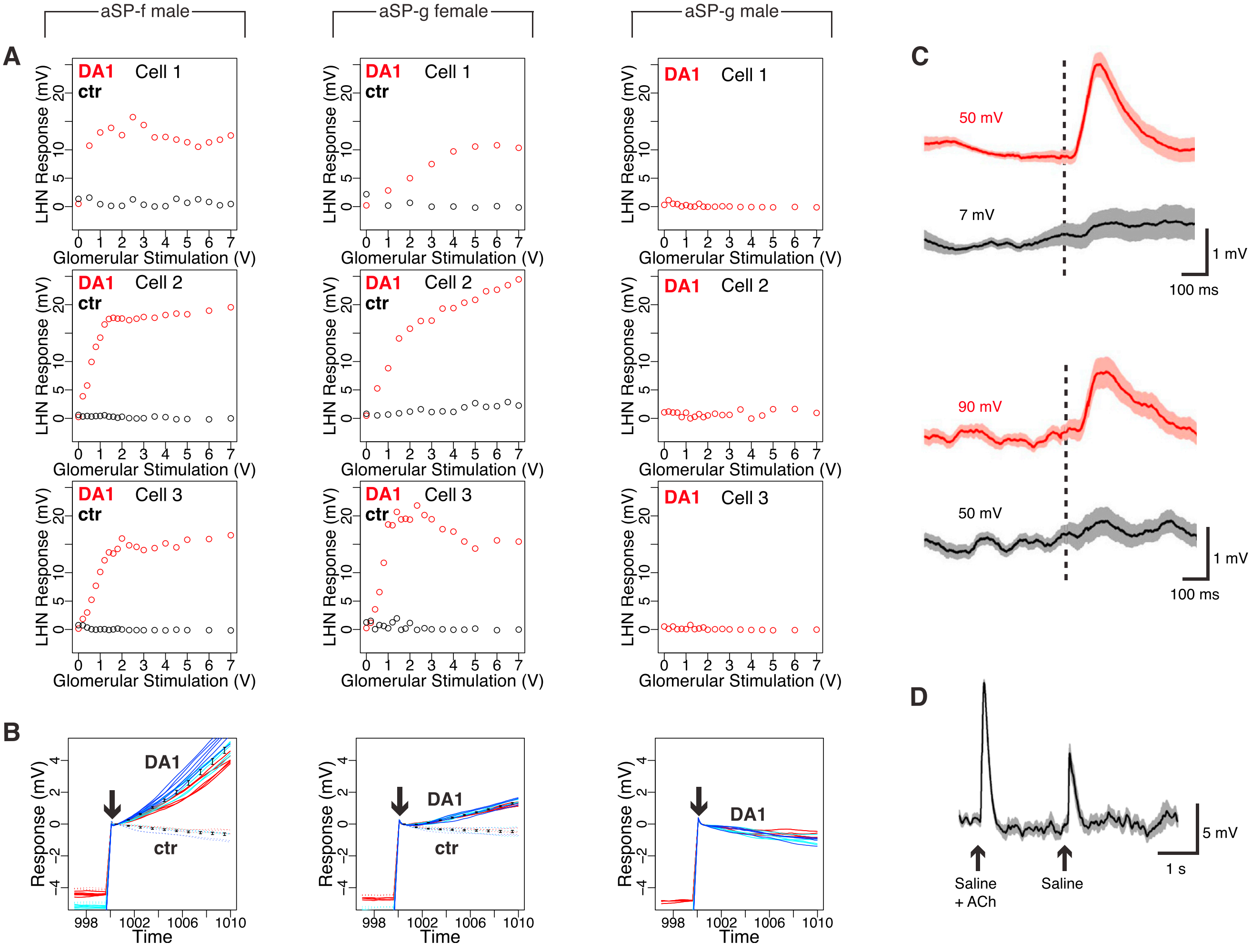

Figure S3. Excitatory Responses in LHNs after DA1 Stimulation, Related to Figure 3

(A and B) depolarizations in LHNs in response to stimulation of DA1 versus control glomerulus (ctr). Panels are arranged in 3 columns, displaying male aSP-f (left), female aSP-g (middle) and male aSP-g (right) neurons. (A) LHN responses to different stimulation intensities in DA1 versus control glomerulus for 3 individual cells (Cell 1–3). Note that no control stimulation was performed in nonresponsive male aSP-g neurons. Note also that aSP-f neurons always fire a spike burst immediately after stimulation but high stimulus intensities produce transient rather than sustained firing (see Figure 3G) and saturating voltage responses (e.g., see Cell 2, left column in (A)). There are several possible explanations including an inability to fire spikes in the face of massive presynaptic input or recruitment of an inhibitory pathway. (B) Voltage traces for DA1 versus control stimulation diverge rapidly after stimulation onset. Three increasing input voltages are displayed for each of 3 cells (red, cyan, blue lines with increasing stimulus intensity, 2–6 traces per stimulus level). Solid lines indicate DA1 stimulation, dotted lines control glomerulus stimulation. The average ± SEM is shown for the DA1 and control stimuli. Arrows label stimulation onset.

(C) Iontophoretic stimulation of DA1 evokes excitatory responses (red) in male aSP-f neurons (top) and female aSP-g neurons (bottom) at low stimulation voltages. Traces are averages of n = 100 individual stimulations at the indicated stimulation voltage (±SEM). Dashed lines mark stimulation onset.

(D) Iontophoretic stimulation of DA1 has both a chemical and an electrical component, as illustrated by a representative postsynaptic response of a male aSP-f neuron to DA1 stimulation (5 V, 20 ms) with 2 mM acetylcholine in saline versus saline only; the responses can be directly compared because they were delivered from each barrel of a double-barreled theta electrode. Traces are averages of n = 10 stimulations (±SEM).

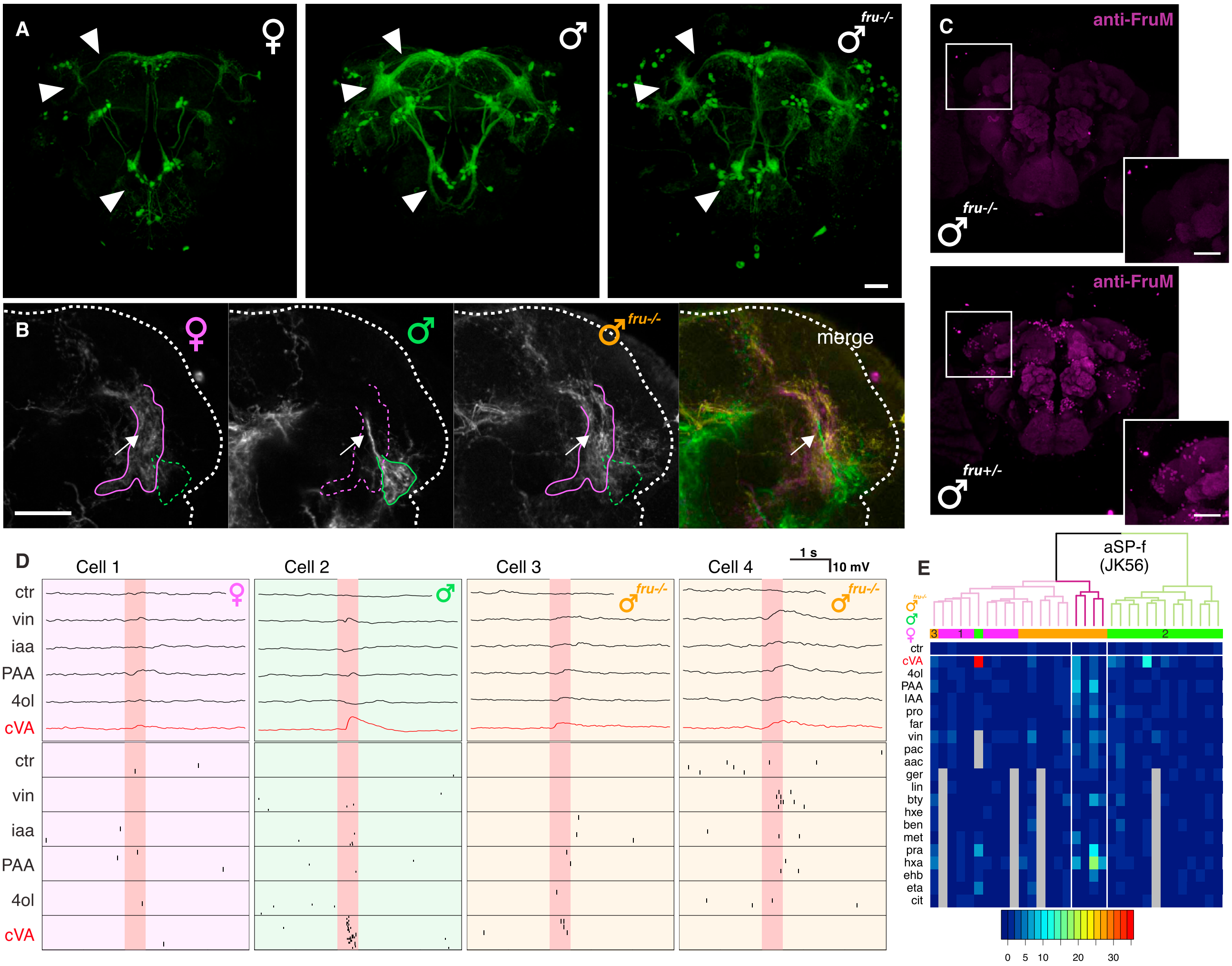

Figure S4. Fru^{M} Is Necessary for Male Morphology of aSP-f Neurons, Related to Figure 4

(A) Male-enlarged or -specific brain regions (arrowheads) are feminized in *fru*$^{-/-}$ males. However, the overall *JK56* expression does not appear completely feminized in *fru*$^{-/-}$ males.

(B) Partial Z projections of the lateral horn of wild-type female (left), wild-type male (middle left) and *fru*$^{-/-}$ male (middle right) *JK56* animals. Male-specific aSP-f tract (arrow) and aSP-f dendrites in the ventral lateral horn (green outline) are absent in females and *fru*$^{-/-}$ males. Female-specific arborizations (magenta outline) are absent from male brains but present in *fru*$^{-/-}$ male brains.

(C) Fru^{M} is absent from the brains of (top) *fru*$^{-/-}$ animals but detectable in the brains of (bottom) *fru*^{F} / *MKRS* control flies.

(D) Physiological data for *JK56* aSP-f neurons. Data for female (Cell 1), male (Cell 2) and *fru*$^{-/-}$ male (Cell 3–4) aSP-f neurons are shown. The top row shows averaged current clamp recordings, the second row shows raster plots for the same neurons.

(E) Mean odor responses of wild-type male, female and *fru*$^{-/-}$ male *JK56* aSP-f neurons displayed as heatmap. Data are organized by a dendrogram of the morphological similarity between each neuron presented at the top of the panel. Dendrograms are split into colored subclusters. Underneath each dendrogram, one row indicates the sex of each neuron. Each column is a single neuron and each row an odorant. Each box represents the color-coded average spiking frequency of a median of 6 odor trials. Grey boxes indicate odorants not tested. Neurons displayed in (D) (Cell 1–3) are highlighted with numbers (1–3) in the first row. Cell 4 is not displayed (unsuccessful registration). All scale bars 25 μm. Pale red bars in (D) mark 500 ms odor presentation.

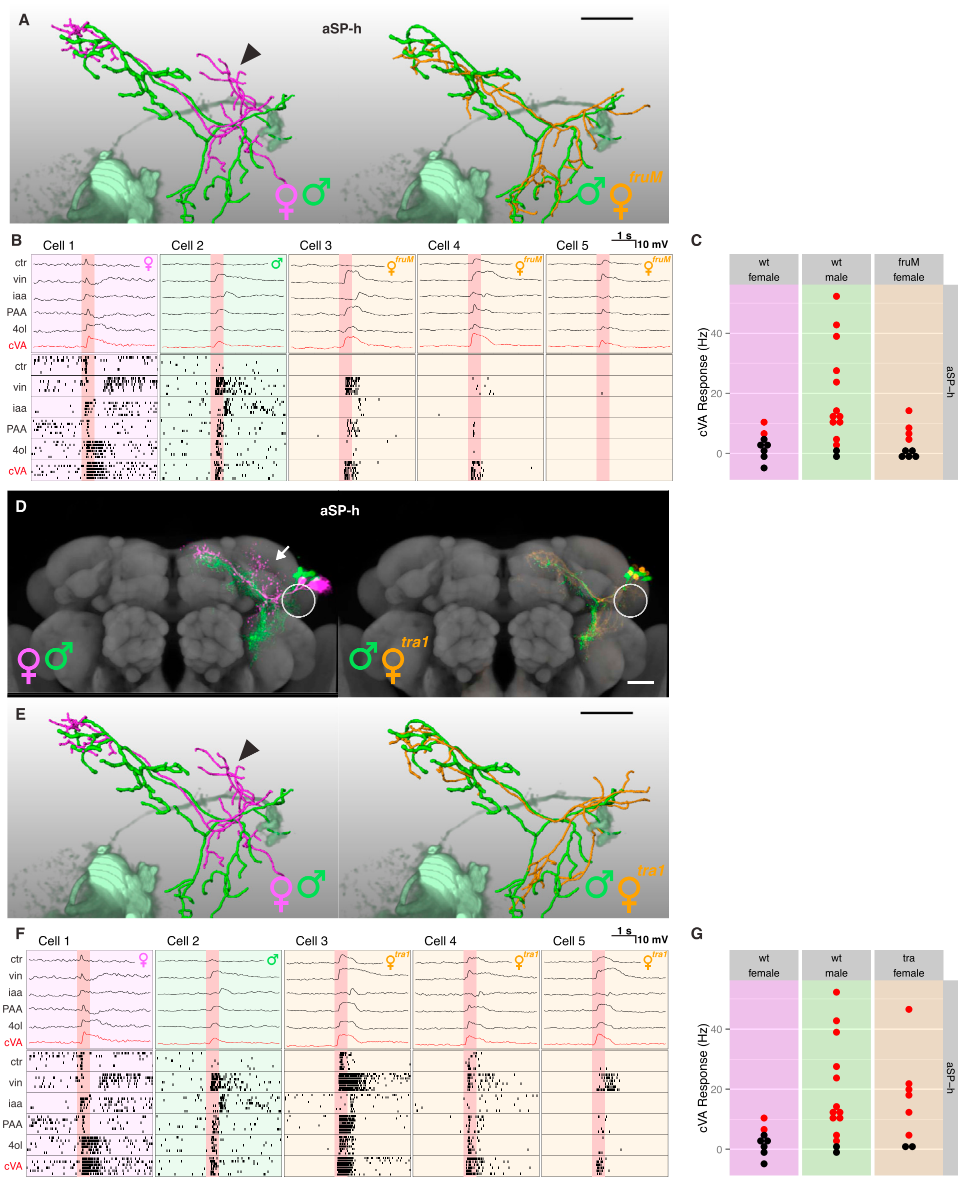

Figure S5. Masculinization of aSP-h Neurons in *fru^M* Females and *tra^1* Clones, Related to Figure 5

(A–C) Transformed aSP-h neurons in *fru^M* females. (A) Single dye-filled and reconstructed female, male and *fru^M* female aSP-h neurons compared with volume rendered DA1 PNs (pale green). Note the male-like overlap of *fru^M* female aSP-h dendrites with DA1 PNs and the missing female-specific dorsal branch (arrowhead). (B) Physiological data for wild-type female, wild-type male and three *fru^M* mutant aSP-h neurons. The top row shows averaged current clamp recordings of each LHN displayed in (A). The second row shows raster plots for the same neurons. (C) Summary of cVA responses. Each dot represents a neuron, significant cVA responses are in red.

(D–G) Transformed *tra^1* female aSP-h neurons. (D) Z projections of female, male and *tra^1* mutant female aSP-h neuroblast clones onto a reference brain with the ventral lateral horn marked with a white circle. Note that transformed *tra^1* female aSP-h clones lack the female-specific dorsal branch (arrow) and are indistinguishable from their male counterpart. (E) Single dye-filled and reconstructed female, male and *tra^1* female aSP-h neurons compared with volume rendered DA1 PNs (pale green). Note the male-like overlap of *tra^1* female aSP-h dendrites with DA1 PNs and the missing female-specific dorsal branch (arrowhead). (F) Physiological data for wild-type female, wild-type male and three *tra^1* mutant aSP-h neurons. The top row shows averaged current clamp recordings of each LHN displayed in (E). The second row shows raster plots for the same neurons. (G) Summary of cVA responses. Each dot represents a neuron, significant cVA responses are in red. See Table S1C for full statistical analysis. All scale bars 25 μm. Pale red bars in (B) and (F) mark 500 ms odor presentation.

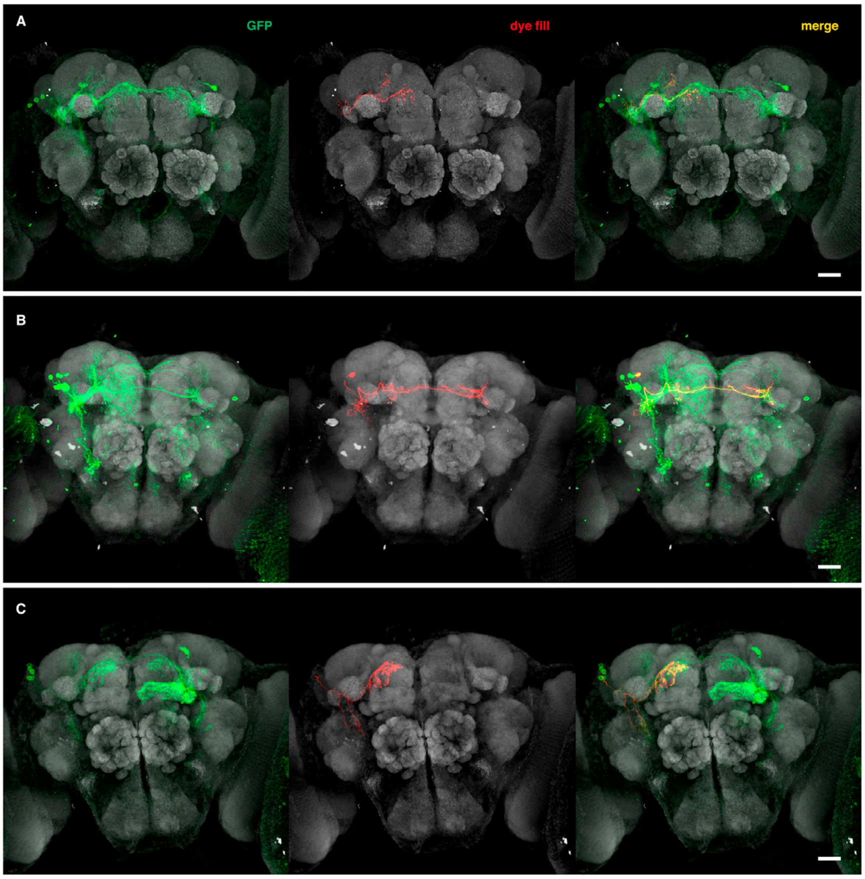

Figure S6. Single Recorded and Dye-Filled LHNs in *tra*[1] Mutant Female Brains, Related to Figure 6

(A–C) Partial Z-projections of *tra*[1] mutant (A) aSP-f, (B) aSP-g and (C) aSP-h MARCM clones (left) and single, dye-filled neurons (middle). Nonregistered (see the Experimental Procedures) nc82-stained brains are shown in gray. Note the presence of several additional single-cell clones in (A) and (B) and a Kenyon cell clone in (C). All scale bars 25 μm.
